# Both stronger and weaker cerebro‐cerebellar functional connectivity patterns during processing of spoken sentences in autism spectrum disorder

**DOI:** 10.1002/hbm.26478

**Published:** 2023-09-09

**Authors:** Jussi Alho, John G. Samuelsson, Sheraz Khan, Fahimeh Mamashli, Hari Bharadwaj, Ainsley Losh, Nicole M. McGuiggan, Steven Graham, Zein Nayal, Tyler K. Perrachione, Robert M. Joseph, Catherine J. Stoodley, Matti S. Hämäläinen, Tal Kenet

**Affiliations:** ^1^ Department of Neurology Massachusetts General Hospital, Harvard Medical School Boston Massachusetts USA; ^2^ Athinoula A. Martinos Center for Biomedical Imaging, Massachusetts General Hospital, Harvard Medical School Boston Massachusetts USA; ^3^ Harvard‐MIT Division of Health Sciences and Technology, Massachusetts Institute of Technology Cambridge Massachusetts USA; ^4^ Department of Radiology Massachusetts General Hospital, Harvard Medical School Boston Massachusetts USA; ^5^ Department of Speech, Language, and Hearing Sciences, and Weldon School of Biomedical Engineering Purdue University West Lafayette Indiana USA; ^6^ Department of Speech, Language, and Hearing Sciences Boston University Boston Massachusetts USA; ^7^ Department of Anatomy and Neurobiology Boston University School of Medicine Boston Massachusetts USA; ^8^ Department of Psychology College of Arts and Sciences, American University Washington DC USA

**Keywords:** autism, cerebellum, functional connectivity, MEG, speech

## Abstract

Cerebellar differences have long been documented in autism spectrum disorder (ASD), yet the extent to which such differences might impact language processing in ASD remains unknown. To investigate this, we recorded brain activity with magnetoencephalography (MEG) while ASD and age‐matched typically developing (TD) children passively processed spoken meaningful English and meaningless Jabberwocky sentences. Using a novel source localization approach that allows higher resolution MEG source localization of cerebellar activity, we found that, unlike TD children, ASD children showed no difference between evoked responses to meaningful versus meaningless sentences in right cerebellar lobule VI. ASD children also had atypically weak functional connectivity in the meaningful versus meaningless speech condition between right cerebellar lobule VI and several left‐hemisphere sensorimotor and language regions in later time windows. In contrast, ASD children had atypically strong functional connectivity for in the meaningful versus meaningless speech condition between right cerebellar lobule VI and primary auditory cortical areas in an earlier time window. The atypical functional connectivity patterns in ASD correlated with ASD severity and the ability to inhibit involuntary attention. These findings align with a model where cerebro‐cerebellar speech processing mechanisms in ASD are impacted by aberrant stimulus‐driven attention, which could result from atypical temporal information and predictions of auditory sensory events by right cerebellar lobule VI.

## Graphical abstract

Cerebellar differences have long been documented in autism spectrum disorder (ASD), yet the extent to which such differences might impact language processing in ASD remains unknown. To investigate this, we recorded brain activity with magnetoencephalography (MEG) while ASD and age‐matched typically developing (TD) children passively processed spoken meaningful English and meaningless Jabberwocky sentences. Using a novel source localization approach that allows higher resolution MEG source localization of cerebellar activity, we found that the functional connectivity for meaningful versus meaningless speech contrast in ASD children was atypically weak between right cerebellar lobule VI and several left‐hemisphere sensorimotor and language regions in a time window toward the end of the sentences, yet atypically strong between right cerebellar lobule VI and primary auditory cortical areas in an earlier time window.

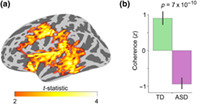

## 1 INTRODUCTION

Children with autism spectrum disorder (ASD) commonly show impairments in speech and language (Mody et al., 2013; Tager‐Flusberg et al., 2005), with deficits in lexical‐semantic processing among the most consistently reported findings (Boucher, 2012; McGregor et al., 2012). While the behavioral manifestations of such impairments in ASD are well documented, the neural mechanisms underlying these impairments are still not fully understood. The cerebellum is known to contribute to language processing via reciprocal connections with the cerebral cortex (Mariën et al., 2014; Schmahmann, 2019) and is one of the most common sites of abnormality in ASD, such that cerebellar dysfunction has been suggested to be crucial in the etiology of ASD (Becker & Stoodley, 2013; D'Mello & Stoodley, 2015; Su et al., 2021; Wang et al., 2014). To date, though, the role of the cerebellum in language processing abnormalities in ASD is relatively unexplored.

In an earlier work, we reported atypical lexical‐semantic processing of spoken sentences in ASD children by comparing neuromagnetic cortical evoked responses to meaningful speech sentences and their meaningless Jabberwocky counterparts during passive listening (Alho et al., 2021). We found an interaction effect where ASD children had weaker responses to meaningful compared with meaningless sentences in the same left temporal and parietal language regions where typically developing (TD) children had stronger responses to meaningful sentences. Importantly, the amplitude of the responses was associated with scores measuring ASD severity and aberrant involuntary attentional shifting in ASD. We interpreted these findings to reflect dysfunctional receptive speech processing in ASD, wherein unattended meaningful speech elicits atypically weak engagement of the language system, whereas unattended meaningless speech, filtered out in TD individuals, engages the language system in ASD through involuntary attention capture. At the time, due to the limitations of magnetoencephalography (MEG) source modeling, our analysis was restricted to cortical language regions.

In the present study, we applied a novel source localization approach that allows higher resolution MEG source localization of cerebellar activity (Samuelsson et al., 2020) to investigate the interactions between the cerebellum and the cortex during the same paradigm. We were interested in the role of the cerebellum because, in addition to its well‐established role in motor control (Fine et al., 2002; Holmes, 1939), it has more recently been demonstrated to be central also to cognitive function, including language processing (Booth et al., 2007; Frings et al., 2006; Jansen et al., 2005; Schmahmann & Sherman, 1998; Stoodley et al., 2010, 2021). More specifically, functional magnetic resonance imaging (fMRI) and positron emission tomography (PET) studies have revealed that, in individuals with left cerebral hemisphere dominance for language, language tasks activate mainly the right posterolateral cerebellum lobules VI, Crus I, and Crus II (Guell et al., 2018; Stoodley & Schmahmann, 2009).

Importantly, although both the right cerebellar lobules VI and VII (which includes Crus I and Crus II) have been implicated in language processing, anatomical diffusion‐weighted MRI and fMRI resting‐state functional connectivity studies have suggested functional differences between these two lobules: while lobule VI is primarily connected with cerebral sensorimotor regions, lobule VII mostly connects with parietal and prefrontal cortices as part of a cognitive circuit (Habas et al., 2009; Krienen & Buckner, 2009; O'Reilly et al., 2010; Salmi et al., 2010; for a review, see Stoodley et al., 2021). In language processing, fMRI (Frings et al., 2006), neuromodulation (Turkeltaub et al., 2016), and lesion studies (Stoodley et al., 2016) have differentiated speech articulation to medial lobule V/VI and more cognitive aspects of language to posterolateral lobules VI/VII. How cerebellar language function might differ in ASD remains unknown.

An additional motivation to investigate the role of the cerebellum in this paradigm stems from the fact that both altered cerebellar circuitry and altered cerebro‐cerebellar functional connectivity have been reported in ASD (Arnold Anteraper et al., 2019; Fatemi et al., 2012; Igelström et al., 2017; Khan, Nair, et al., 2015; Noonan et al., 2009; Oldehinkel et al., 2019; Olivito et al., 2017; Verly et al., 2014). Some fMRI studies have reported a pattern of simultaneously decreased canonical cerebro‐cerebellar connectivity within established networks and increased non‐canonical cerebro‐cerebellar connectivity between regions that are not typically correlated (Khan, Nair, et al., 2015; Noonan et al., 2009). Similar findings of reduced network integration (i.e., underconnectivity within neurotypical networks) and differentiation (i.e., overconnectivity with regions outside neurotypical networks) have been reported also in cortico‐cortical functional connectivity in ASD (Fishman et al., 2014; Fishman et al., 2015; Keown et al., 2017; Rudie et al., 2012; Shih et al., 2011). Atypical reduction in fMRI resting state functional connectivity between the cerebellum and cerebral language networks in ASD has also been reported (Arnold Anteraper et al., 2019; Verly et al., 2014). For example, Verly et al. (2014) showed disrupted connectivity between right Crus I and left cerebral cortical language regions yet preserved connectivity between the cerebral cortical language regions in ASD, thus providing indirect evidence of the role of the cerebellum in language processing abnormalities in ASD.

Given the relevance of the cerebellum to both language processing and ASD etiology, we wanted to investigate how cerebellar activity and cerebro‐cerebellar functional connectivity during the processing of spoken sentences are affected by the presence versus absence of lexical‐semantic information in ASD children, using the same set of meaningful and meaningless sentences as in Alho et al. (2021). Based on the results from our earlier work, we hypothesized that the ASD group would show weaker responses in the right cerebellar lobules VI and VII to the meaningful speech versus meaningless Jabberwocky condition than the TD group. We further hypothesized that the functional connectivity in the meaningful speech versus meaningless Jabberwocky condition between right cerebellar lobule VI/VII and left cerebral cortical language regions would be weaker in the ASD group. We tested these hypotheses by analyzing MEG data collected from children and adolescents diagnosed with ASD (*N* = 25) and age‐matched TD (*N* = 26) individuals ages 7–17 years.

## 2 MATERIALS AND METHODS

### 2.1 Participants

In total, 30 individuals between ages 7 and 17 diagnosed with autism spectrum disorder (ASD), and 35 age‐matched typically developing (TD) individuals participated in the study. Five individuals (two ASD and three TD) were excluded from the analyses due to poor MEG data quality, and four individuals (two ASD and two TD) were excluded due to distorted cerebellum reconstructions due to insufficient MRI data quality. To better match the resulting groups on nonverbal IQ (NVIQ), another five (one ASD and four TD) individuals were excluded, resulting in a final sample of 25 ASD and 26 TD participants. Sample characteristics are summarized in Table 1 (for histogram of age distribution of the sample, see Figure S1).

**TABLE 1:** Characterization of the participants.

	ASD (*N* = 25, 3 females)			TD (*N* = 26, 5 females)			
	*N*	Mean (SD)	Range	*N*	Mean (SD)	Range	*p*‐value
Age	25	13.8 (3.1)	7–17	26	13.6 (3.1)	7–17	.85
NVIQ	25	104.4 (13.3)	74–136	26	110.5 (11.6)	93–130	.10
VIQ	25	101.5 (16.9)	61–131	26	110.7 (14.1)	71–140	.04
ADOS‐2	25	12.0 (3.8)	7–20	—	—	—	—
SRS	24	93.4 (23.6)	30–128	—	—	—	—
SCQ	24	17.8 (6.4)	5–31	24	3.5 (3.2)	0–12	<.001
ASPS	19	26.0 (7.1)	13–38	24	35.6 (3.9)	27–40	<.001
ICSS	21	8.0 (3.7)	2–15	20	10.5 (3.7)	3–17	.05
SCSS	21	9.0 (3.0)	1–14	19	10.4 (3.6)	4–17	.19

*Note*: The *p*‐values are from two‐sample *t*‐tests (two‐tailed) for the difference in means between the ASD and TD groups.

Abbreviations: ADOS‐2, Autism Diagnostic Observation Schedule—Second Edition; ASPS, Auditory Sensory Profile Score; ICSS, NEPSY‐II Inhibition Contrast Scaled Score; NVIQ, Nonverbal IQ; SCQ, Social Communication Questionnaire; SCSS, NEPSY‐II Switching Contrast Scaled Score; SRS, Social Responsiveness Scale; VIQ, Verbal IQ.

All participants had normal hearing and confirmed hearing the stimuli well in each ear before the onset of the paradigm. Participants with ASD had a prior clinical diagnosis of ASD and met a cutoff of ≥15 on the Social Communication Questionnaire, Lifetime Version, and met clinical criteria on the Autism Diagnostic Observation Schedule (ADOS; Lord et al., 2000) administered by trained research personnel who had established inter‐rater reliability. Individuals with autism‐related medical conditions (e.g., Fragile‐X syndrome, tuberous sclerosis) and other known risk factors (e.g., gestation <36 weeks) were excluded from the study.

All TD participants were below threshold on the Social Communication Questionnaire and were confirmed to be free of any neurological or psychiatric conditions, and of substance use for the past 6 months, via parent‐reports and self‐reports. Verbal IQ (VIQ) and NVIQ were assessed with the Kaufman Brief Intelligence Test—II (Kaufman, 2004) for 21 ASD and 16 TD participants, and with the Differential Ability Scales—II (Elliot, 2007) for 4 ASD and 10 TD participants. Handedness information was collected using the Dean Questionnaire (Piro, 1998). The Social Responsiveness Scale parent report (SRS‐2; Constantino & Gruber, 2012), which was designed as a quantitative measure of autism‐related symptoms, was collected from all participants and used as an ASD severity score.

Additionally, a subset of participants completed the Sensory Profile Questionnaire (Brown & Dunn, 2002). For the correlations with MEG data, we used the sum score of the eight questions of the Auditory section of the Sensory Profile, referred hereon as ASPS (Auditory Sensory Profile Score). Lastly, a subset of participants completed the INN (Inhibition–Naming), INI (Inhibition–Inhibition), and INS (Inhibition–Switching) sections of the NEPSY‐II. From this, the Inhibition Contrast Scaled Score (ICSS) measures inhibition of attention, and the Switching Contrast Scaled Score (SCSS) measures attentional switching. The ICSS and SCSS scores range from 1 to 19. All research was conducted in compliance with the Massachusetts General Hospital Institutional Review Board (MGH IRB), and all participants were consented in accordance with the Declaration of Helsinki and the approved protocol. Parents provided informed consent according to protocols approved by the MGH IRB. Assent was obtained from children ages 13 and above.

### 2.2 Stimuli and paradigm

**FIGURE 1:**
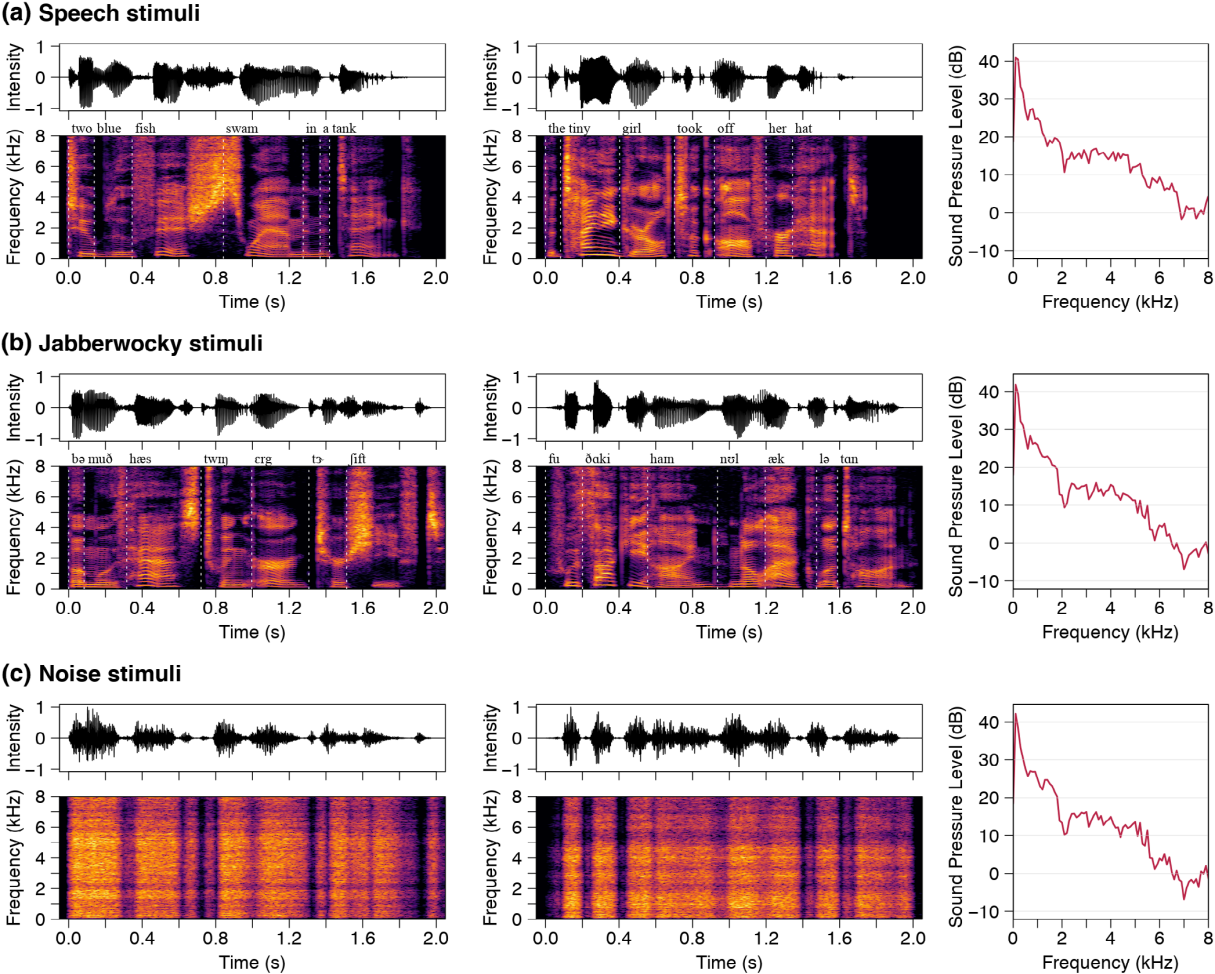
Stimulus acoustics. Stimulus waveforms (top), spectrograms (bottom), and their long‐term average spectra (right) are shown for the (a) Speech, (b) Jabberwocky, and (c) Noise conditions. Transcriptions of the sentences are written above the spectrograms. Noise stimuli were modulated with the spectra and amplitude envelopes of stimuli in the Jabberwocky condition. The participants watched a movie with the sound off while the sentences were presented in random order via earphones.

The stimuli consisted of auditorily presented spoken English sentences (“*Two blue fish swam in a tank*” and “*The tiny girl took off her hat*”) and matched spoken Jabberwocky sentences (where words were replaced by pseudowords; Figure 1). The English sentence stimuli (referred hereon as “Speech”) were taken from the IEEE sentences (Anon, 1969). The Jabberwocky sentences (referred hereon as “Jabberwocky”) were taken from a corpus derived from the IEEE sentences (Perrachione et al., 2015). The Jabberwocky sentences were created by re‐arranging the phonemes from the Speech sentences while adhering to the phonotactic rules of English. The corresponding Speech and Jabberwocky sentences had the same number of phonemes and syllables, with no difference in the duration of individual phonemes (*F*_1,32_ = 1.38, *p* = .25), and no condition × phoneme interaction (*F*_22,32_ = 0.79, *p* = .71). Further, the phoneme and biphone positional probabilities (Vitevitch & Luce, 2004) of the Jabberwocky pseudowords were matched with the words in the Speech sentences (phonemes: *F*_1,27_ = 0.004, *p* = .95; biphones: *F*_1,27_ = 0.87, *p* = .36). Low‐level acoustic cues between the Speech and Jabberwocky stimuli were controlled by having the same, highly trained phonetician (T.K.P.) produce the sentences.

In addition, amplitude‐modulated noise was used as control stimuli. Two noise stimuli were created by generating stationary noise stimuli with the spectrum shaped to match the long‐term frequency content of the Jabberwocky stimuli, and then amplitude‐modulating them with the broadband envelopes of the two Jabberwocky sentences.

The stimuli were presented binaurally through insert earphones (Etymotic, Elk Grove Village, IL) at 75 dB sound pressure level. To direct attention away from the stimuli, the participants watched a muted video of their choosing and were instructed to ignore the sound stimuli. This design was chosen to minimize the impact of the ability of the participant to maintain attention during the paradigm.

Each stimulus was presented 40 times in the paradigm, totaling 80 Speech, 80 Jabberwocky, and 80 Noise trials (with some exceptions; see Figure S2 for the exact number of trials per condition per participant). The trials counts did not differ significantly between the groups (Wilcoxon rank‐sum test: *z* = 0.24, *p* = .81). The stimuli were presented in random order with a 700–800 ms randomly varying inter‐stimulus interval. The paradigm additionally included two categories of sinewave speech sentences (60 repetitions each) presented randomly along with the Speech, Jabberwocky, and Noise stimuli, which are not discussed here. The paradigm was presented in three runs, each lasting about 6 min.

### 2.3 Structural MRI data acquisition and processing

T1‐weighted magnetization‐prepared rapid gradient echo (MPRAGE) structural images were acquired using a 3 T Siemens Trio MRI scanner (Siemens Medical Systems, Erlangen, Germany) with a 32‐channel head coil (in‐plane resolution 1 × 1 mm; slice thickness 1.3 mm, TR 2530 ms; TI 1100 ms; TE 3.39 ms; flip angle 7°). Cortical reconstructions and parcellations were generated using the FreeSurfer software (documented and available at http://surfer.nmr.mgh.harvard.edu/).

### 2.4 MEG data acquisition

The MEG data were acquired with a whole‐head 306‐channel VectorView neuromagnetometer (MEGIN Oy, Finland) inside a magnetically shielded room (IMEDCO, Switzerland). The 306 channels are arranged in 102 sensor triplets with two orthogonal planar gradiometers and one magnetometer. The signals were band‐pass filtered at 0.1–200 Hz prior to sampling at 1000 Hz. The position of the head was continuously recorded during the data acquisition using four head position indicator (HPI) coils attached to the scalp (Uutela et al., 2001). Locations of the HPI coils, three anatomical landmarks (nasion and auricular points), and multiple additional scalp surface points were digitized using a Fastrak digitizer (Polhemus) to allow coregistering the MEG and MRI data. Additionally, electrocardiography (ECG) and electro‐oculography (EOG) were recorded to detect heartbeats, and eye movements and blinks, respectively. At the end of each subject measurement session, 5 min of empty room data were recorded without the subject present to estimate the noise covariance matrix for MEG source analysis.

### 2.5 MEG data preprocessing

First, bad MEG channels were detected using visual inspection. We then applied temporal Signal Space Separation (tSSS; Taulu & Kajola, 2005; Taulu & Simola, 2006), as implemented in the MNE‐Python Maxwell filtering routine, to compensate for head movements during the recording as well as to reduce artifacts originating from both external sources outside the MEG sensor array and from the space between the brain and the MEG sensor array. The average head movements (±standard deviation) during the recording for the TD and ASD groups were 0.17 ± 0.12 mm and 0.25 ± 0.26 mm, respectively, with no statistically significant difference between the groups (two‐sample *t*‐test: *t* = 1.40, *p* = .17). We quantified the head movements by converting the six head motion parameters (three translation and three rotation) to millimeters and combining them into a time series of head movement (by taking the Euclidean norm at each sample). We used the default tSSS parameters (inside expansion order of 8, outside expansion order of 3, subspace correlation limit of 0.98, and raw data buffer length of 10 s) and applied fine calibration and cross‐talk correction data specific to the recording site.

We then applied independent component analysis (ICA) to the tSSS‐processed data to reduce systematic physiological artifacts, such as eye blinks and heartbeats. More specifically, we used FastICA (Hyvärinen, 1999) to decompose MEG signals into maximally independent components (ICs). The ICA decomposition was estimated on band‐pass filtered (1 Hz highpass, 30 Hz lowpass) data. Segments where signal amplitude exceeded 4000 fT/cm and 4000 fT on the gradiometers and magnetometers, respectively, were excluded from the estimation. The ICs corresponding to ECG or EOG activity were identified based on Pearson correlation and visual inspection of scalp topographies corresponding to each of the components. The average (±standard deviation) number of ICs excluded in the TD and ASD groups were 3.12 ± 0.37 and 3.22 ± 0.46, respectively.

### 2.6 Source estimation

The cerebral cortical surface reconstruction was made using FreeSurfer (Fischl, 2012). The cerebellar cortical surface reconstruction was made using the methods described in Samuelsson (2021). The cerebellar and cerebral surface reconstructions were decimated using an ~2‐mm grid spacing. The forward solution was computed using a single‐compartment boundary‐element model (BEM; Hämäläinen & Sarvas, 1987). The inner skull surface triangulations were generated from the MRI data using the watershed algorithm.

The inverse analysis was done in MNE‐python (Gramfort et al., 2013). The cerebellar estimates were found using the methods described in Samuelsson (2021). The Standardized LOw Resolution brain Electromagnetic TomogrAphy (sLORETA) approach (Pascual‐Marqui, 2002) with a loose source orientation constraint (Lin, Belliveau, et al., 2006) of 0.2 and depth weighting (Lin, Witzel, et al., 2006) of 0.8 was used as the inverse method. Compared with its non‐normalized counterpart minimum‐norm estimate (MNE), the noise‐normalized sLORETA estimate has been shown to provide a better source localization of subcortical sources such as the cerebellum (Samuelsson, 2021). The noise covariance matrix used in the inverse operator was estimated from the empty room data.

### 2.7 Delineating cerebellar regions of interest

**FIGURE 2:**
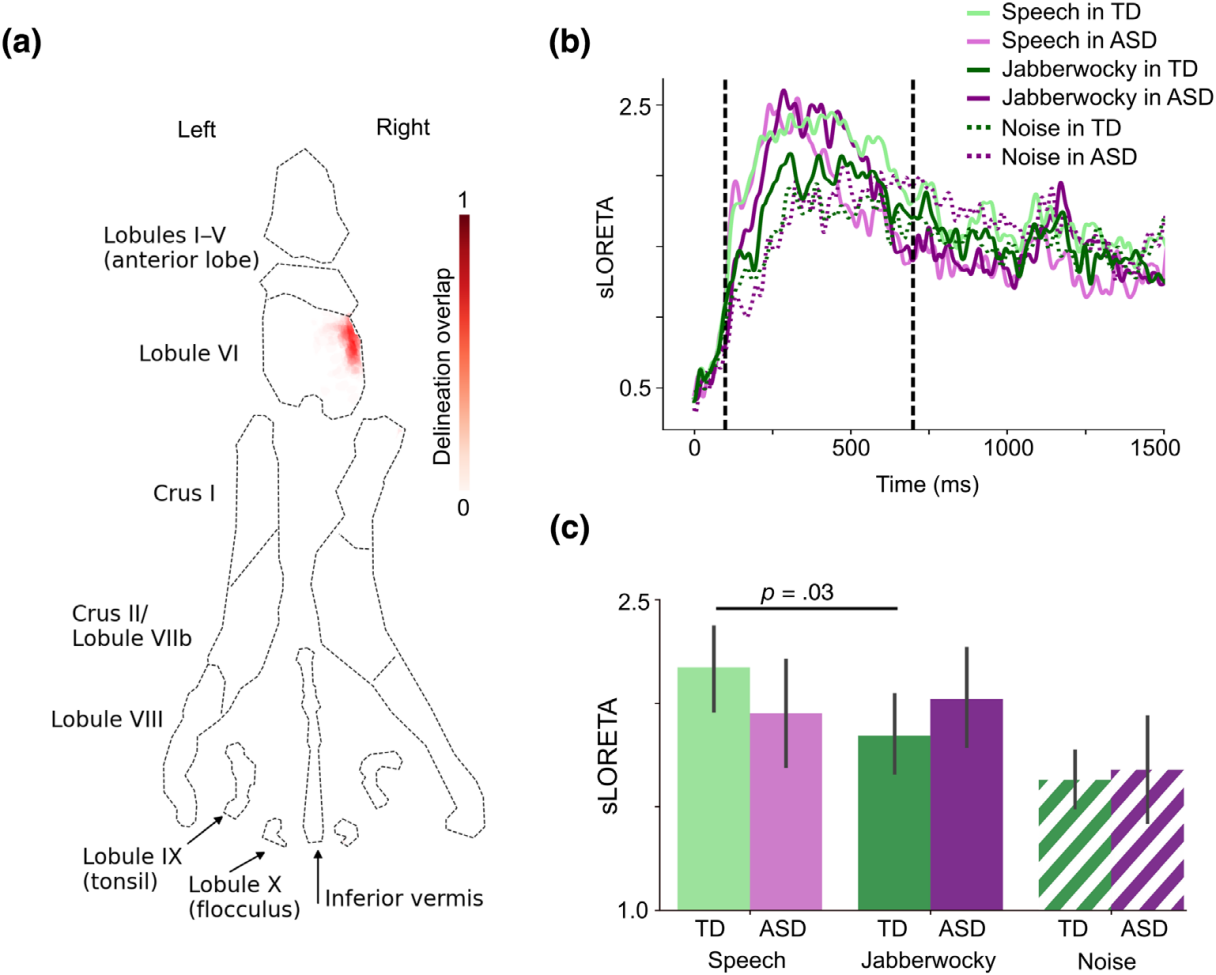
ERFs in right cerebellar lobule VI. (a) Flatmap representation of the cerebellum with probability map of the right lobule VI delineation overlap across participants (*N* = 51). (b) sLORETA time courses for each group and condition from the region depicted in a. Vertical dashed lines show the time window of interest (100–700 ms after stimulus onset). (c) Bar graph of group means averaged within the time window in B. The *p*‐value is from a paired‐samples *t*‐test (two‐tailed). Error bars represent standard error of the mean. See Figure S3 for the cortical ERFs.

We defined functional regions of interest (ROIs) within lobule VI, Crus I, and Crus II in both hemispheres. For each participant, we selected 50 vertices within the anatomical atlas region (~1% of the vertices within the anatomical region) with the largest mean sLORETA estimates of the event‐related fields (ERFs) within 0–1500 ms after stimulus onset. The ERFs were derived by averaging epochs across combined Speech and Jabberwocky conditions. Before averaging, the epochs were low‐pass filtered at 30 Hz, baseline‐corrected using a 200‐ms prestimulus period, and the epoch counts between the Speech and Jabberwocky conditions were equalized. Figure 2a shows the overlapping probability of the ROI within right lobule VI across participants visualized on a cerebellar flatmap representation (Samuelsson, 2021).

### 2.8 Delineating cerebral ROIs

**FIGURE 3:**
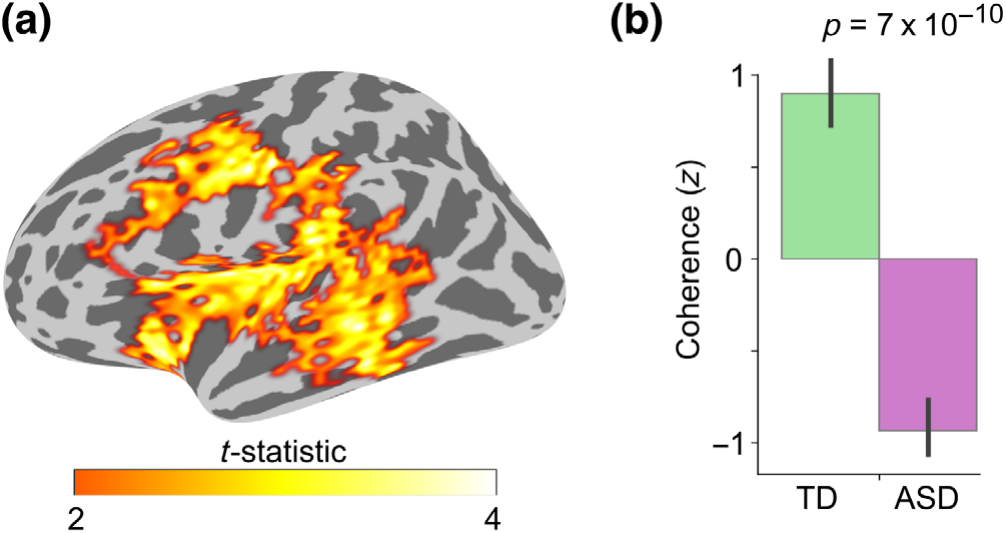
Group difference in the Speech versus Jabberwocky functional connectivity with the right cerebellar lobule VI seed. (a) Spatial extent of the spatio‐temporal group difference cluster depicted on inflated left lateral cerebral hemisphere. The spatial cluster representation was derived by collapsing the temporal dimension by selecting the time point of the largest group difference for each vertex. (b) Bar graph of group means with *p*‐value from two‐sample *t*‐test (two‐tailed). Coherence values were averaged within the whole spatio‐temporal cluster, corrected for NVIQ, and the residuals were *z*‐scored. Error bars represent standard error of the mean.

**FIGURE 4:**
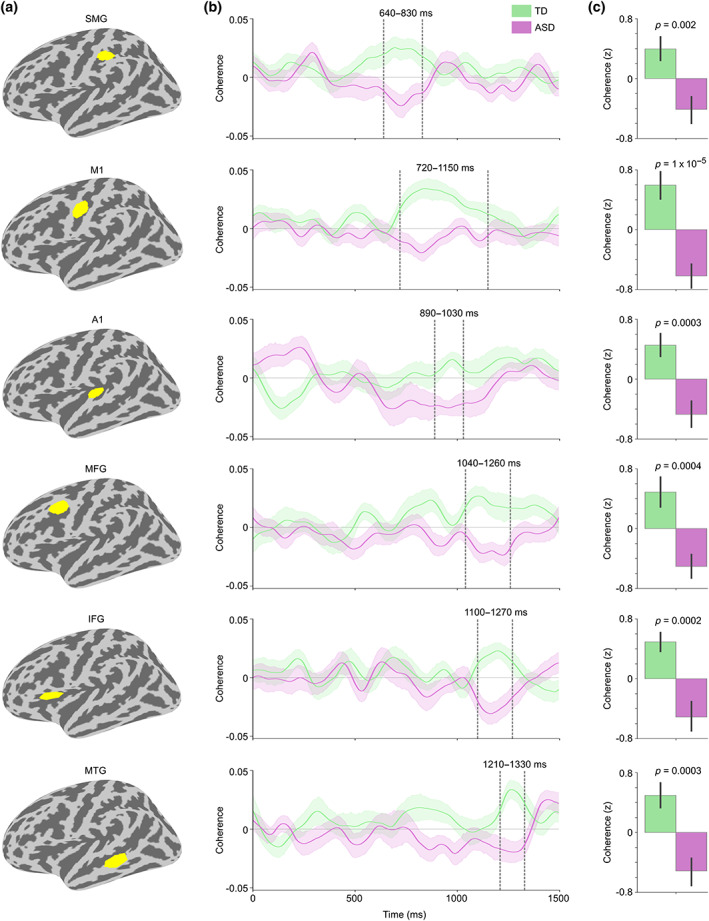
Coherence between right cerebellar lobule VI and cortical ROIs for Speech versus Jabberwocky in TD and ASD groups. (a) ROIs from top to bottom: supramarginal gyrus (SMG), primary motor cortex (M1), primary auditory cortex (A1), middle frontal gyrus (MFG), inferior frontal gyrus (IFG), and middle temporal gyrus (MTG). (b) Right lobule VI seed coherence time courses from the ROIs in TD and ASD groups. Vertical dashed lines show time windows of significant group difference in the permutation test (also marked above the time windows). The ROI time courses are arranged from the earliest to the latest significant group difference. Shaded areas around the group mean time courses indicate standard error of the mean. (c) Bar graph of group means averaged within the time windows in b with *p*‐values from two‐sample *t*‐tests (two‐tailed). Coherence values were corrected for NVIQ and the residuals were *z*‐scored. Error bars represent standard error of the mean.

Cerebral ROIs were defined by thresholding the significant right lobule VI seed connectivity group difference cluster (Figure 3a) at *t* = 3.5 (corresponding to *p* = .001) and selecting the largest surviving cluster (spatially adjacent vertices) within an anatomical atlas region. We chose six anatomical regions known to be related to auditory and language processing (Hickok & Poeppel, 2007; Rauschecker & Scott, 2009) from FreeSurfer cortical parcellations (Desikan et al., 2006; Destrieux et al., 2010): primary auditory cortex/superior temporal gyrus (A1; “transversetemporal” and “superiortemporal” atlas labels), middle temporal gyrus (MTG; “middletemporal”), supramarginal gyrus (SMG; “S_postcentral”), primary motor cortex (M1; “precentral”), middle frontal gyrus / premotor cortex (MFG; “S_precentral‐inf‐part”), and inferior frontal gyrus (IFG; “parsopercularis,” “parstriangularis,” and “parsorbitalis”). Figure 4a shows the resulting cerebral ROIs.

Because the ROIs were defined based on group differences, as a control, we also defined ROIs based solely on anatomical parcellations (Desikan et al., 2006; Destrieux et al., 2010). We selected the following six auditory and language regions (Hickok & Poeppel, 2007; Rauschecker & Scott, 2009) from the parcellations: “transversetemporal” (A1), middle third of “S_temporal_sup” (MTG), inferior third of “S_postcentral” (SMG), inferior third of “S_central” (M1), superior half of “S_precentral‐inf‐part” (MFG), and frontal half of “S_circular_insula_sup” (IFG). The divisions of the original parcellation regions were done using the “split_label” function of MNE Python, which finds the original parcellation region's principal eigen‐axis on the spherical surface, projects the coordinates of all the vertices within the region onto this axis and divides them at regular spatial intervals (https://mne.tools/stable/generated/mne.split_label.html). Sulci were favored in the delineation of the anatomical ROIs for the supplemental analysis (the capital “S” in the beginning of the parcellation labels stands for “Sulcus”) due to the selective sensitivity of MEG to tangentially oriented sources, located predominantly on the walls of the sulci (Ahlfors et al., 2010). Figure S5A shows the final anatomical ROIs.

### 2.9 Cerebellar event‐related fields

We first defined epochs of MEG data starting 200 ms before and ending 1500 ms after the stimulus onset for the Speech, Jabberwocky, and Noise conditions. Before epoching, the data were low‐pass filtered at 30 Hz. The epoched data were baseline corrected by subtracting the mean amplitude in a 200‐ms pre‐stimulus period from the signals and ERFs were obtained by averaging across the epochs. Before averaging, the epoch counts were equalized between the three conditions (when necessary). The ROI‐specific ERFs were obtained by averaging the sLORETA source estimates of the ERFs across the vertices within the ROI.

### 2.10 Seed‐based functional connectivity

We computed seed‐based functional connectivity in each condition (Speech, Jabberwocky, Noise) from the cerebellar ROIs to the cerebral cortex. The seed time courses were obtained by averaging across the sLORETA source estimates of the ERFs across the vertices within each of the ROIs (i.e., left lobule VI, right lobule VI, left Crus I, right Crus I, left Crus II, right Crus II). Time‐frequency decomposition of the resulting seed time courses and the time courses of all cortical vertices was done using continuous wavelet transform with complex Morlet wavelets (each spanning seven cycles) in a frequency range of 4–30 Hz and a time window of 0–1500 ms with respect to stimulus onset.

Connectivity was quantified as phase synchrony across epochs for every time and frequency point using coherence and phase‐locking value (PLV) (Lachaux et al., 1999). The results for each frequency were binned into theta (4–8 Hz), alpha (8–14 Hz), and beta (14–30 Hz) bands, and subsequently averaged within each frequency band. Before the connectivity computation, the epoch counts were equalized between the Speech, Jabberwocky, and Noise conditions within participant. The analysis was performed using MNE‐Python (Gramfort et al., 2013) with the help of the Massachusetts Life Sciences Center (MLSC) Compute Cluster.

Cortical regions where there were significant group differences were determined using cluster‐based permutation statistics (Maris & Oostenveld, 2007). To this end, the data of each participant was morphed to a FreeSurfer average cortical representation with 4098 vertices per hemisphere (Fischl et al., 1999) and temporally decimated to 10 ms steps. The medial wall, limbic lobe, and occipital lobe were excluded from the analysis and the test was run separately for the left and right hemispheres using a two‐tailed *t*‐test and cluster‐forming threshold of *t* = 2.0 (corresponding to *p* = .05) and 5000 permutations. Clusters were formed based on spatial and temporal adjacency. Cluster‐level statistics were calculated by summing the *t*‐statistics within the formed cluster. The contrast conditions (Speech vs. Jabberwocky, Speech vs. Noise, and Jabberwocky vs. Noise) were derived by first averaging the connectivity values within the frequency bands and calculating the difference between the resulting connectivity time courses. For the correlation analyses, we selected the peak connectivity value for each participant within the time window showing significant group difference. Correlations were tested using Pearson (product–moment) correlation coefficient and differences in within‐group correlations were assessed using Fisher's *r*‐to‐*z* transformation.

### 2.11 Directionality of the functional connectivity

We computed nonparametric Granger causality (Dhamala et al., 2008a, 2008b) to estimate directionality of the functional connectivity. We used a single Hanning taper frequency transformation for 4–30 Hz with 4 Hz steps of the epoched time courses with a 250‐ms sliding window and 25 ms steps between 0 and 1500 ms after stimulus onset. Granger causality scores were calculated for each window using nonparametric spectral matrix factorization (Dhamala et al., 2008b). Before the connectivity computation, the epoch counts were equalized between the Speech, Jabberwocky, and Noise conditions within participant. The analysis was performed using FieldTrip toolbox (Oostenveld et al., 2011). Group differences were tested using two‐sample *t*‐tests (two‐tailed). Correlations were tested using Pearson (product–moment) correlation coefficient and differences in within‐group correlations were assessed using Fisher's *r*‐to‐*z* transformation.

## 3 RESULTS

### 3.1 Cerebellar event‐related fields

Since our first set of results using this paradigm were obtained by analyzing event‐related fields (ERFs) in the cerebral cortex (Alho et al., 2021), we began by analyzing ERFs in the cerebellar ROIs. Even though the group difference in NVIQ was not statistically significant (see Table 1), given the trend toward a difference, we chose to adjust the brain measures for NVIQ in all between‐group comparisons.

The ERFs in all ROIs were the most prominent in a time window of ~100–700 ms after stimulus onset. In this time window, the ERFs were significantly stronger for Speech versus Jabberwocky in the TD group in right lobule VI (paired‐samples *t*‐test: *t* = 2.25, *p* = .03, *d* = 0.33; Figure 2). No differences were found in the other ROIs. As the strongest group differences in the ERFs in the cerebral cortex in our earlier work were found in a later time window of ~1000–1500 ms after the sentence onset (Alho et al., 2021), we tested the cerebellar ERFs in this time window, too, but did not find any significant group differences in any ROI. Therefore, for the remaining analyses, we focused on the language ROI identified in right lobule VI.

As the present sample overlaps with, but is not identical to, the sample in Alho et al. (2021), we tested the differences in ERFs also in the cerebral cortex, to ensure the prior results are replicated in this sample. Indeed, we found stronger ERFs in the ASD group for Jabberwocky versus Speech between 1000 and 1500 ms after sentence onset in the same left temporal ROI (paired‐samples *t*‐test: *t* = 2.81, *p* = .01, *d* = 0.51; ANOVA group × condition interaction: *F* = 3.24, *p* = .04, $\eta_{p}^{2}=0.08$; Figure S3A) and parietal ROI (paired‐samples *t*‐test: *t* = 3.79, *p* = .001, *d* = 0.51; ANOVA group × condition interaction: *F* = 9.11, *p* = .004, $\eta_{p}^{2}=0.16$; Figure S3B) that showed the most significant differences in Alho et al. (2021). Furthermore, similarly to the ERFs in the right cerebellar lobule VI, the ERFs in the parietal ROI were stronger for Speech versus Jabberwocky in the TD group in the earlier 100–700 ms time window (paired‐samples *t*‐test: *t* = 2.48, *p* = .02, *d* = 0.40; ANOVA group × condition interaction: *F* = 10.8, *p* = .002, $\eta_{p}^{2}=0.18$; Figure S3B).

### 3.2 Functional connectivity between right lobule VI and cerebral cortex

Next, we conducted a seed‐based functional connectivity analysis using the cerebellar ROIs as seeds. With the ROI in right lobule VI as the seed, we found a significant group difference in the alpha band (8–14 Hz) coherence in an extensive spatio‐temporal cluster between ~600 and 1400 ms after stimulus onset in the left cerebral hemisphere (Figure 3a; cluster *p*‐value = .004).

A post hoc two‐sample *t*‐test revealed significantly stronger coherence for Speech versus Jabberwocky in the TD compared with the ASD group (*t* = 7.65, *p* = 7 × 10^−10^, *d* = 2.18; Figure 3b). Significant group differences were also observed when testing the conditions separately (Speech vs. Noise: *t* = 5.81, *p* = 5 × 10^−7^, *d* = 1.66; Jabberwocky vs. Noise: *t* = −3.66, *p* = 6 × 10^−4^, *d* = −1.05; Figure S4). We also used phase‐locking value (PLV) as an alternative functional connectivity measure and got similar results (Speech vs. Jabberwocky: *t* = 4.87, *p* = 1 × 10^−5^, *d* = 1.39). No significant group differences in functional connectivity were found in the right cortical hemisphere with the right lobule VI seed or in either hemisphere with any other seeds.

We then analyzed the functional connectivity between right cerebellar lobule VI and the left cerebral hemisphere in greater detail by defining specific ROIs with significant group differences (Figure 4). The earliest group difference was found in the supramarginal gyrus (SMG; 640–830 ms; *t* = 3.27, *p* = .002, *d* = 0.93), the latest in the middle temporal gyrus (MTG; 1210–1330 ms; *t* = 3.93, *p* = .0003, *d* = 1.12), and the most significant group difference was in the primary motor cortex (M1; 720–1150 ms; *t* = 4.88, *p* = 1 × 10^−5^, *d* = 1.39). Group differences in the other ROIs were as follows: in the primary auditory cortex (A1; 890–1030 ms; *t* = 3.89, *p* = .0003, *d* = 1.11), the middle frontal gyrus/premotor cortex (MFG; 1040–1260 ms; *t* = 3.76, *p* = .0004, *d* = 1.08), and the inferior frontal gyrus (IFG; 1100–1270 ms; *t* = 4.11, *p* = .0002, *d* = 1.17). The group differences remained significant also when using ROIs defined solely based on anatomical parcellations (see Figure S5).

### 3.3 Directionality of the functional connectivity between right lobule VI and each of the cortical ROIs

**FIGURE 5:**
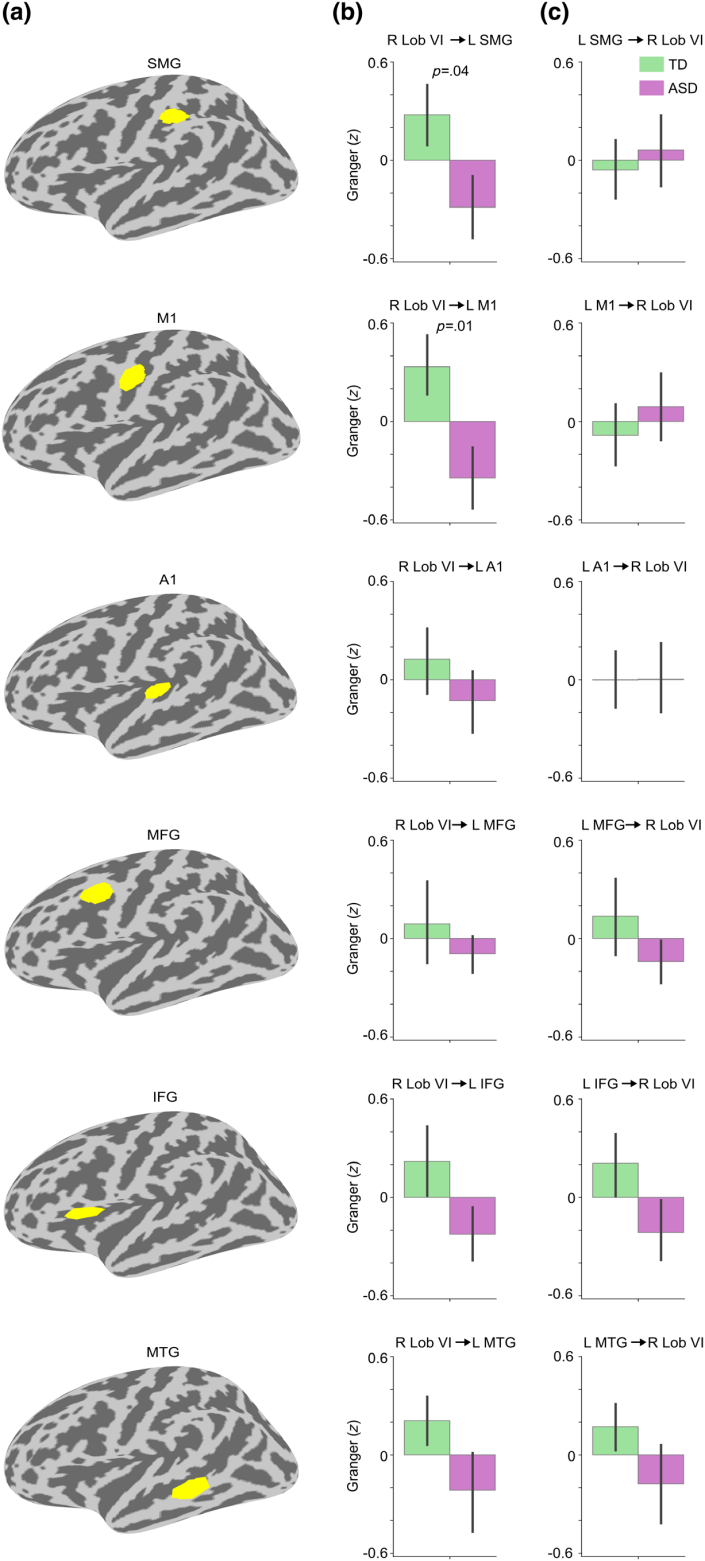
Directionality of the Speech versus Jabberwocky connectivity between right lobule VI and cortical ROIs. (a) The cortical ROIs depicted on left hemisphere inflated surface. The ROIs are arranged based on the latency of the group difference in the right lobule VI seed coherence (from the earliest to the latest; see Figure 4). (b) Bar graphs of group means for the directed connection from right lobule VI to the ROIs. (c) Bar graphs of group means for the directed connection from the ROIs to right lobule VI. In b and c, the direction of the connectivity is shown on top of the graphs. For significant group differences, the *p*‐value from the two‐sample *t*‐test (two‐tailed) is shown on top of the bar graph. The Granger causality was estimated using 8–12 Hz center frequencies and sliding window center points matching the ROI‐specific group difference time windows (see Figure 4) rounding the beginning down and the end up to the nearest 50 ms. For example, the Granger causality sliding window center points for SMG were 600–850 ms. Error bars around the mean represent standard error of the mean. Granger causality values were corrected for NVIQ and the residuals were *z*‐scored. L, left; R, right.

To gain additional insight into the nature of the functional connectivity differences, we conducted post‐hoc tests to estimate whether these ROI‐specific functional connections showed directionality effects by using nonparametric Granger causality analysis (Figure 5). We found significantly stronger Granger causality for directed connectivity from right lobule VI to left SMG (*t* = 2.06, *p* = .04, *d* = 0.59) and the left M1 (*t* = 2.52, *p* = .01, *d* = 0.72) in the TD compared with the ASD group. Furthermore, the connectivity between right lobule VI and left M1 showed a significant direction × group ANOVA interaction (*F* = 7.39, *p* = .009, $\eta_{p}^{2}=0.13$). The direction × group ANOVA interaction for the connectivity between right lobule VI and left SMG did not reach significance (*F* = 2.57, *p* = .11, $\eta_{p}^{2}=0.05$). The other functional connections did not show significant group differences or direction × group interactions.

### 3.4 Early directional functional connectivity between right lobule VI and left A1

**FIGURE 6:**
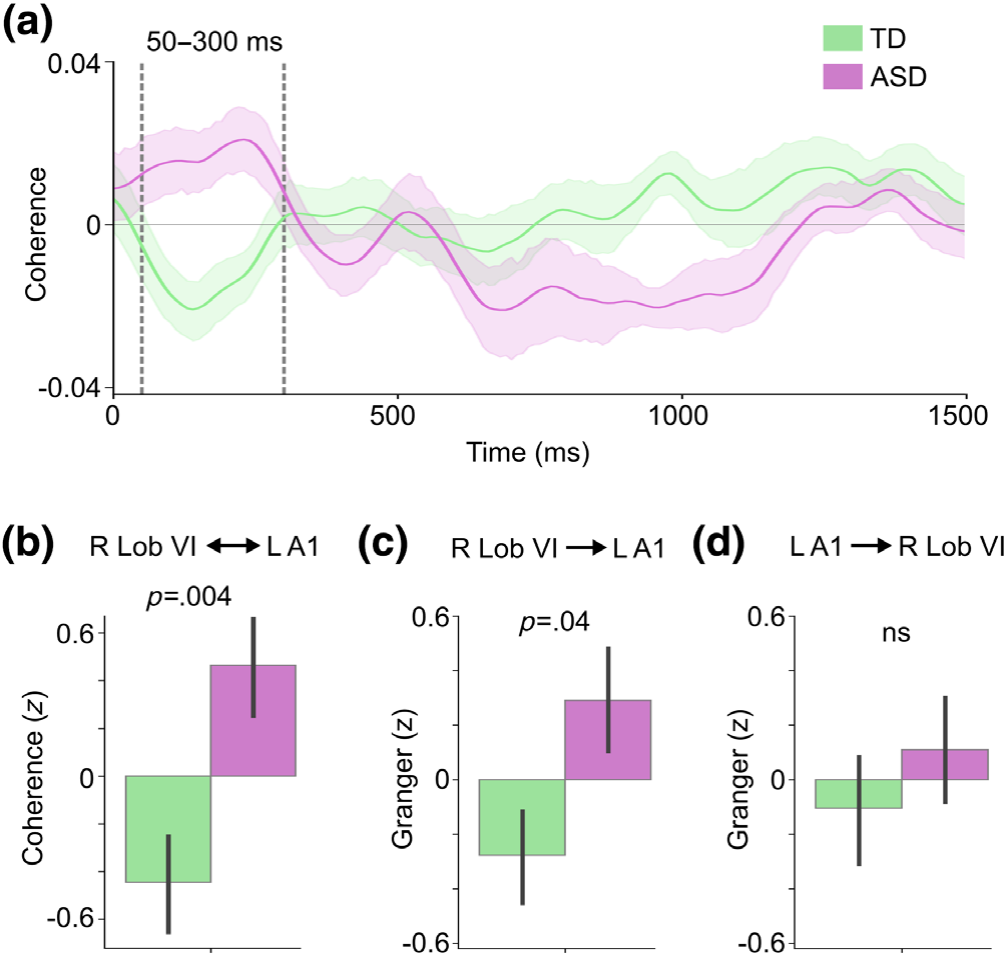
Group difference in the Speech versus Jabberwocky functional and effective connectivity between right lobule VI and left primary auditory cortex (A1). (a) Right lobule VI seed coherence time courses from the A1 ROI in TD and ASD groups. Vertical dashed lines show the 50–300 ms time window of interest. Shaded areas around the group mean time courses indicate standard error of the mean. (b) Bar graph of group means averaged within the time window of interest in A. (c) Bar graph of group means for the directed connectivity from right lobule VI to left A1 with *p*‐value from two‐sample *t*‐test (two‐tailed). (d) Bar graph of group means for the directed connectivity from left A1 to right lobule VI. In b–d, the direction of the connectivity is shown on top of the graphs. Granger causality in c and d was estimated using 8–12 Hz center frequencies and 125–300 ms sliding window center points relative to stimulus onset. Values in b–d were corrected for NVIQ and the residuals were *z*‐scored. The *p*‐values are from two‐sample *t*‐test (two‐tailed). Error bars around the mean represent standard error of the mean. L, left; ns, not significant; R, right.

As noted before, the largest ERFs in the cerebellum were in an early time window (~100–600 ms). Also, as can be seen in Figure 4, there was an apparent early group difference in the coherence between right lobule VI and left A1 in the opposite direction, with seemingly greater functional connectivity in the ASD group. To further analyze this time window, we conducted a post‐hoc test by averaging the individual coherence values within a 50–300 ms after‐stimulus‐onset time window (Figure 6a). Two‐sample *t*‐test (two‐tailed) revealed significantly stronger Speech versus Jabberwocky coherence in the ASD compared with the TD group (*t* = 3.06, *p* = .004, *d* = 0.88; Figure 6b).

To estimate the directionality of the early connectivity between right lobule VI and left A1, we again used nonparametric Granger causality analysis. The results showed significantly stronger effective connectivity in the ASD compared with the TD group from right lobule VI to left A1 (*t* = 2.07, *p* = .04, *d* = 0.68; Figure 6c) but not from left A1 to right lobule VI (*t* = 0.75, *p* = .46, *d* = 0.22; Figure 6d). To test whether the functional connectivity in the early (50–300 ms) and later (600–1400 ms) time windows were associated with one another, we calculated the correlation between these measures, but did not find significant correlations in either group.

### 3.5 Correlation of ERFs and functional connectivity with age

Considering that language processing undergoes maturation in the age range (7–17) of the present sample (Skeide & Friederici, 2016), as well as the growing number of studies showing abnormal maturational trajectories of various neuroimaging metrics in ASD (for review, see Edgar, 2020) both from our group (Khan, Michmizos, et al., 2015; Mamashli et al., 2018, 2021) and others (Alaerts et al., 2015; Luna et al., 2007; Wallace et al., 2010), we assessed the effect of age on both the ERFs and the functional connectivity in each group.

We first tested the correlation between the ERFs and age. The ERFs in right lobule VI did not show significant correlations with age in either group, nor were there significant differences in the within‐group correlations. Then, we tested whether right lobule VI seed connectivity correlated with age. We again did not find any significant within‐group correlations with age, nor any significant group differences between the within‐group correlations, suggesting relatively similar maturational trajectories in the ERFs and the functional connectivity between right lobule VI and left cerebral cortex for both the TD and ASD groups. Figure S6 shows the results from all the correlation tests between age and the right lobule VI seed connectivity in all ROIs.

### 3.6 Correlation of ERFs and connectivity measures with behavioral scores

To determine whether the atypical cerebellar activity or functional connectivity for Speech versus Jabberwocky in the ASD group was related to participant characteristics, we calculated correlations between the neurophysiological measures and four behavioral scores: ASD severity (SRS), a measure of auditory sensory processing (ASPS), and two measures of attention—inhibition (ICSS) and switching (SCSS).

**FIGURE 7:**
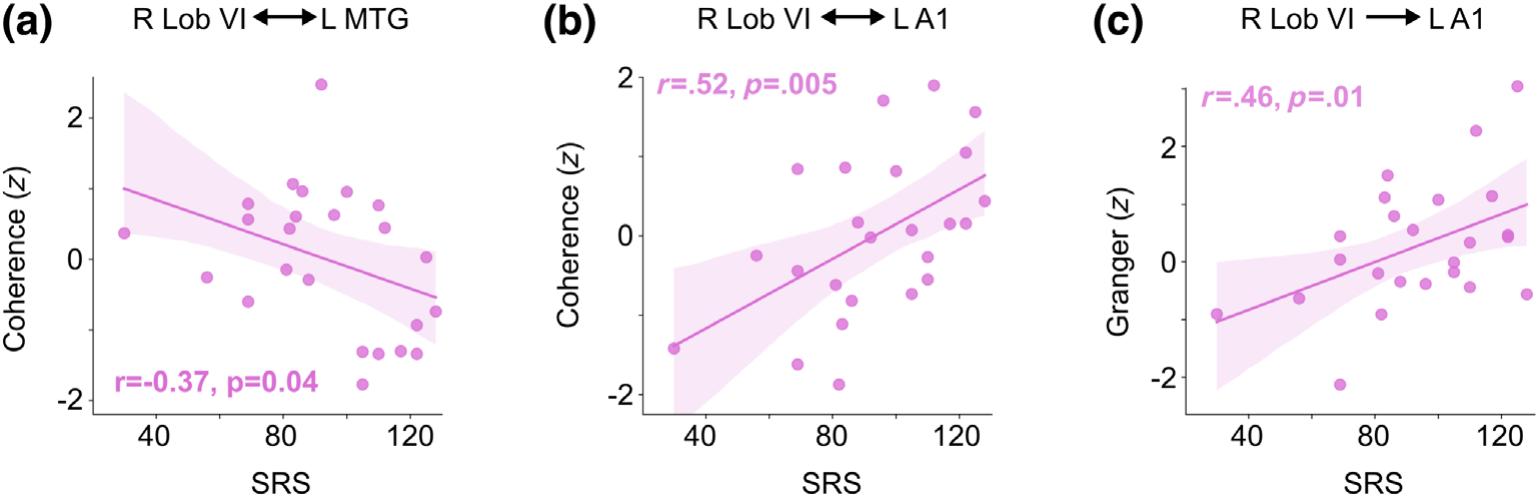
Correlation between ASD severity and Speech versus Jabberwocky connectivity between right lobule VI and left cortical ROIs. (a) Scatter plot of SRS total scores against Speech versus Jabberwocky coherence between right lobule VI and left MTG within the 1210–1330 ms time window (see Figure 4). (b) Scatter plot of SRS total scores against Speech versus Jabberwocky coherence between right lobule VI and left A1 within the 50–300 ms time window (see Figure 6). (c) Scatter plot of SRS total scores against Speech versus Jabberwocky Granger causality scores. Granger causality was estimated using 8–12 Hz center frequencies and 125–300 ms sliding window center points relative to stimulus onset. The shaded areas around the regression line encompass the 95% confidence interval for the correlation. Correlation coefficient (*r*) and *p*‐value from Pearson correlation test (one‐tailed) are shown in each plot.

We first tested whether any of the neurophysiological measures were associated with ASD severity. While the ERFs did not show significant correlations with the SRS, the Speech versus Jabberwocky coherence between right lobule VI and left MTG within the 1040–1260 ms group difference time window showed a significant negative correlation with the SRS scores in the ASD group (*r* = −.37, *p* = .04, Figure 7a), indicating that the more severe the ASD, the weaker the Speech versus Jabberwocky coherence. Further, the Speech versus Jabberwocky coherence in the 50–300 ms after stimulus onset between right lobule VI and left A1 was positively correlated with the SRS scores in the ASD group (*r* = .52, *p* = .005; Figure 7b), indicating that the more severe the ASD, the stronger the Speech versus Jabberwocky coherence.

Similarly, the Granger causality scores for the directed Speech versus Jabberwocky connectivity from right lobule VI to left A1 within 300 ms after stimulus onset correlated positively with the SRS scores within the ASD group (*r* = .46, *p* = .01; Figure 7c), meaning stronger effective connectivity from right lobule VI to left A1 was correlated with more severe ASD.

**FIGURE 8:**
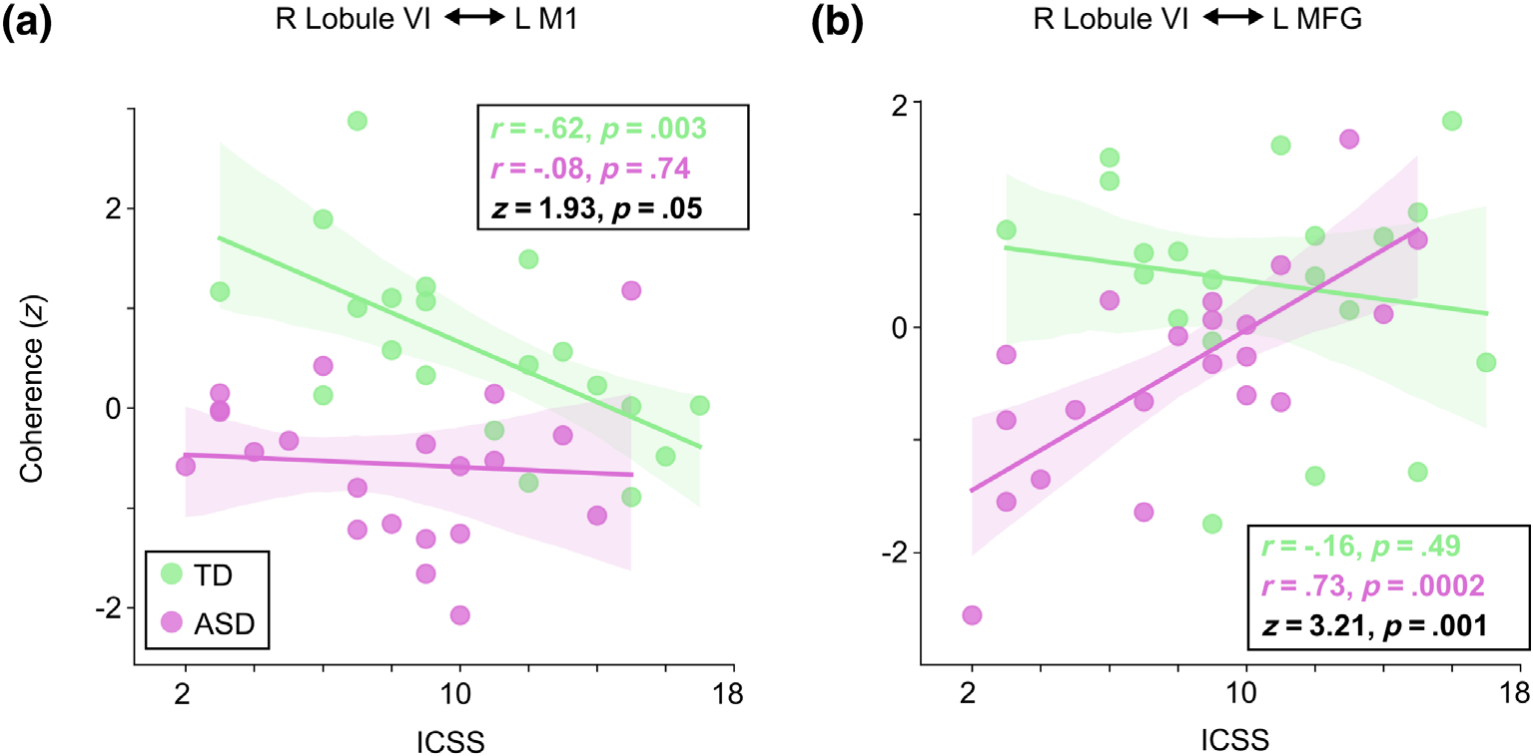
Correlation between attentional inhibition (ICSS) and Speech versus Jabberwocky functional connectivity between right lobule VI and left cortical ROIs. a) Scatter plot of ICSS against Speech versus Jabberwocky coherence between right lobule VI and left M1 within the 720–1150 ms group difference time window (see Figure 4). b) Scatter plot of ICSS against Speech versus Jabberwocky coherence between right lobule VI and left MFG within the 1040–1260 ms group difference time window. The coherence values were adjusted for NVIQ and the residuals were *z*‐scored. The shaded areas around the regression line encompass the 95% confidence interval for the correlation. Correlation coefficient (*r*) and *p*‐value from Pearson correlation test (two‐tailed) for the within‐group correlations as well as Fisher *r*‐to‐*z* transformed *z*‐scores and *p*‐values for the difference between the within‐group correlations are shown in each plot.

The ASPS scores, measuring auditory processing abnormalities, did not show any significant correlations with any of the brain measures. The same was true for the SCSS scores, which measure attentional switching. In contrast, we found a strong negative correlation between the ICSS, which measures attentional inhibition, with the Speech versus Jabberwocky coherence between right lobule VI and left M1 within the 720–1150 ms group difference time window in the TD group (*r* = −.62, *p* = .003; Figure 8a), indicating that poorer ability to inhibit involuntary attentional capture is associated with stronger Speech versus Jabberwocky coherence. This correlation also differed significantly (*z* = 1.93, *p* = .05) from the correlation within the ASD group, which was not statistically significant. In contrast, the ICSS correlated positively with the Speech versus Jabberwocky coherence between right lobule VI and left MFG within the 1040–1260 ms time window in the ASD group (*r* = .73, *p* = .0002, Figure 8b), indicating that poorer ability to inhibit involuntary attentional capture is associated with weaker Speech versus Jabberwocky coherence. This correlation also differed significantly between the groups (*z* = 3.21, *p* = .001).

## 4 DISCUSSION

We used MEG to study functional connectivity between language‐relevant cerebellar and cerebral cortical regions during passive processing of meaningful versus meaningless spoken sentences in ASD children. In evoked responses, TD children showed selectivity to meaningful versus meaningless sentences in right cerebellar lobule VI, while there was no such differentiation in ASD children. Functional connectivity results varied by time window. In a later time window (~600–1400 ms), we found atypically weak functional connectivity in ASD children for meaningful versus meaningless sentences between right lobule VI and extensive left‐hemisphere sensorimotor and language regions. Granger causality suggested that the group differences were driven primarily by directional connectivity from right lobule VI to left supramarginal gyrus (SMG) and primary motor cortex (M1) between 600 and 1150 ms, while the later connectivity with left frontal and temporal regions aligned with a bidirectional pattern of reduced connectivity. For left primary auditory cortex (A1) in an earlier time window (50–300 ms), group differences were reversed in direction, showing increased functional connectivity in ASD children to meaningful versus meaningless sentences, and these differences were again driven by directional connectivity from right lobule VI to left A1. Thus, for cortical areas that are considered lower in cortical hierarchy and where connectivity from the cerebellum peaked earlier, group differences were driven by directional connectivity from the cerebellum to cortex, whereas later connectivity between right lobule VI and left frontal and temporal regions was more reciprocal in nature. Importantly, the functional connectivity measures showing significant group differences correlated with behavioral measures of ability to inhibit involuntary attention and ASD severity.

### 4.1 Both stronger and weaker functional connectivity between right cerebellar lobule VI and left cerebral cortex in ASD

As noted above, we found that the direction of functional connectivity group differences depended on the cortical region of interest. Early into the stimulus sentences, functional connectivity during meaningful versus meaningless sentences was stronger in ASD children between right lobule VI and left A1, whereas later into the sentences it was weaker between right lobule VI and left sensorimotor and language regions in the temporal, parietal, and frontal cortices.

While initially these results might seem contradictory, there are prior indications in the literature that functional connectivity abnormalities in ASD are dependent on topographical principles of organization in brain networks. Indeed, besides agreeing with previous fMRI studies showing weakened resting‐state functional connectivity in ASD between the cerebellum and cortical language regions (Arnold Anteraper et al., 2019; Verly et al., 2014), our findings are also congruent with results showing that functional connectivity was simultaneously decreased within canonical and increased within non‐canonical cerebro‐cerebellar networks in ASD (Khan, Nair, et al., 2015; Noonan et al., 2009).

These findings can also be interpreted in the context of linguistic processing. It is possible that the weaker functional connectivity between the right lobule VI and left‐hemisphere language regions in ASD at the later time window (~600–1400 ms) into the meaningful sentences could reflect a disruption in higher‐order construction of linguistic meaning. This possible interpretation is consistent with both our earlier work with the same stimuli (Alho et al., 2021) as well as other work revealing a monotonic increase in activity in left‐hemisphere language regions over the course of a meaningful sentence, yet no increase in activity for jabberwocky sentences or word‐lists, implying that the largest difference in the processing of meaningful versus meaningless speech stimuli is around the final words of a sentence (Fedorenko et al., 2016).

At a broader level, our findings are compatible with a model of atypical network organization in ASD with reductions in both within‐network integration and between‐network differentiation (Fishman et al., 2014, 2015; Keown et al., 2017; Rudie et al., 2012; Shih et al., 2011). In our results, weakened functional connectivity for meaningful versus meaningless sentences between right lobule VI and left‐hemisphere sensorimotor and language regions in ASD reflects reduced network integration. The atypically increased functional connectivity for meaningful versus meaningless sentences between right lobule VI and left A1 could be interpreted as reduced network differentiation. A1, while critical for auditory speech processing, is not functionally specialized for lexical‐semantic analysis of speech, especially at the observed early latencies, since semantic processing takes place only >300 ms after stimulus onset (Friederici, 2011). We discuss the interpretation of these results in detail in the following sections.

### 4.2 Cerebellar temporal processing and predictive internal models

While cerebellar involvement in language processing is well‐established (Booth et al., 2007; Frings et al., 2006; Jansen et al., 2005; Stoodley et al., 2010, 2021), its exact role remains unclear. A recent review gave support to a general modulatory, instead of functionally specialized, cerebellar contribution to language function (Vlasova et al., 2022). This general modulation function could be related to temporal processing (Buhusi & Meck, 2005; Coull et al., 2011; Ivry et al., 2002; Wiener et al., 2010). In speech perception, it has been proposed that cerebellar temporal processing prepares cerebral cortical regions for the subsequent perceptual integration of sensory information by providing a representation of the speech signal temporal structure and thus guiding predictive allocation of attention (Schwartze & Kotz, 2016).

Such temporal‐processing‐based preparatory function may be associated with a broader cerebellar role in generating predictive internal models (Courchesne & Allen, 1997; Ito, 1970; Manto et al., 2012; Popa & Ebner, 2019; Wolpert et al., 1998) that could enable more efficient preparation for the acquisition and analysis of incoming sensory information in speech perception. Internal forward models have been commonly referred to in the context of sensorimotor integration, which is crucial in speech production but also subserves speech perception (Liebenthal & Möttönen, 2018; Rauschecker & Scott, 2009; Skipper et al., 2017). Sensorimotor integration is mediated by the dorsal auditory stream, involving left parietal and frontal motor regions (Hickok & Poeppel, 2007; Rauschecker & Scott, 2009). More recently, dorsal stream function has also been implicated in the time‐dependent combination of linguistic elements into syntactic representations during sentence comprehension (Bornkessel‐Schlesewsky & Schlesewsky, 2013).

Although speech processing models have mainly focused on the cortical dorsal stream in generating and maintaining internal forward models (Hickok & Poeppel, 2007; Rauschecker & Scott, 2009), there is extensive evidence supporting the ability of the cerebellum to generate forward models (Imamizu et al., 2000; Kawato, 1999; Popa & Ebner, 2019). Note that lobule VI, showing atypical functional connectivity with left cerebral regions in ASD in our results, has been implicated as part of a sensorimotor cerebro‐cerebellar circuit, connecting with the motor cortex (Hoover & Strick, 1999; Salmi et al., 2010).

Our finding of weakened directional connectivity in ASD from right lobule VI to left SMG and M1 in the meaningful versus meaningless sentences could therefore reflect atypical cerebro‐cerebellar speech processing in ASD where, triggered by stimulus‐driven attention, precise temporal information linguistic element relations provided by right lobule VI promotes temporally specific predictions about upcoming sensory events, thus facilitating time‐dependent building of syntactic structure of the speech sentence. Moreover, the late functional connectivity between right lobule VI and left inferior frontal gyrus (IFG) and middle temporal gyrus (MTG) toward the end of the sentence could reflect a subsequent step where the representation of the temporal and syntactic structure is conveyed to facilitate semantic unification (Hagoort & Indefrey, 2014). A dysfunction in such a cerebro‐cerebellar speech processing mechanism could underlie impairments in lexical‐semantic processing of language in ASD. Importantly, these group differences in coherence were found in the alpha band rhythm, which has been associated with both temporal processing (Klimesch, 2012) and communication in large‐scale networks (Palva & Palva, 2011), thus further supporting our interpretation. Abnormalities in the alpha band rhythm in ASD have been reported also by several previous studies (Alho et al., 2023; Edgar et al., 2015; Khan et al., 2013; Mathewson et al., 2012; Murias et al., 2007).

### 4.3 Correlation of the cerebro‐cerebellar functional connectivity with ASD severity

Both the early increased and later decreased functional connectivity during meaningful versus meaningless sentences between right lobule VI and left cerebral cortex correlated with ASD severity. The decreased connectivity between the right lobule VI and left MTG toward the end of the sentence stimuli correlated negatively with the SRS scores (Figure 7a), indicating that weaker functional connectivity during meaningful speech is associated with more severe ASD. Left MTG has been associated with mapping sound to meaning, serving as a computational interface for accessing widely distributed conceptual‐semantic representations (Hickok & Poeppel, 2007), and along with left IFG, has been also linked with semantic integration in sentence‐level processing (Hagoort & Indefrey, 2014). The correlation of decreased functional connectivity between language‐relevant cerebellar and cerebral regions with ASD severity has also been reported in fMRI resting state studies (Arnold Anteraper et al., 2019; Verly et al., 2014).

The increased early (50–300 ms) connectivity between right lobule VI and left A1 correlated positively with the SRS scores (Figure 7b), such that stronger functional connectivity during meaningful speech was associated with more severe ASD. Similarly, the stronger directional connectivity from right lobule VI to left A1 positively correlated with the SRS scores (Figure 7c). Together, these correlations demonstrate the relevance of the observed functional connectivity deviations to the ASD phenotype.

### 4.4 Correlation of cerebro‐cerebellar functional connectivity with attentional inhibition

Our results showed that the behavioral scores measuring inhibition of attentional capture correlated negatively with functional connectivity during meaningful versus meaningless sentences between right lobule VI and left M1 in the TD group (Figure 8a). In contrast, the ASD group showed strong positive correlation of the same scores with the same functional connectivity contrast between right lobule VI and left MFG (Figure 8b). Corroborating our earlier work (Alho et al., 2021), these correlations imply aberrant attentional orienting in ASD, wherein unattended semantically meaningless Jabberwocky, filtered out in TD, engages the receptive language system in ASD.

Considering that all the implicated regions are associated with motor (or sensorimotor) processing, a link can be drawn between the attention and motor systems in auditory speech perception. It has been postulated that the sensory expectation or prediction generated by an internal forward model (i.e., efference copy) could be understood as selective attentional gain applied to the expected sensory features of a stimulus (Hickok et al., 2011).

### 4.5 Potential contribution of the cerebellum to detail‐focused sensory processing style in ASD

The group difference in functional connectivity between right lobule VI and left A1 at early latencies (<300 ms; Figure 6) could reflect detail‐focused processing of the meaningful speech sentences in the ASD group. Such bias toward local over global processing of sensory stimuli is well‐documented in ASD, especially in vision, but also in the auditory domain (Bouvet et al., 2014; Mottron et al., 2006; Plaisted et al., 1998; Wang et al., 2015). The significant interaction between group and condition in functional connectivity is congruent with the proposed different sensory processing styles between the groups: while in TD, the socially relevant meaningful speech is processed globally and the meaningless Jabberwocky more locally, the opposite seems to hold in ASD. It has been argued that this atypical perceptual organization in ASD might relate to a deficit in the temporal synthesis of sensory information; that is, slower integration of local features into a global percept, which would particularly impact dynamic perception, such as auditory speech perception, in which global percepts are built up sequentially over time (Van Der Hallen et al., 2015; Robertson & Baron‐Cohen, 2017). The finding of increased directional connectivity from the right lobule VI to the left primary auditory cortex could therefore reflect an enhanced effort of the cerebellum to facilitate the (access to details through) sequencing and integration of the incoming dynamic sensory information of the meaningful speech in ASD. Furthermore, the finding is consistent with the notion that the local–global perceptual style in ASD has a low‐level processing origin rather than resulting from modulation of early sensory processing by higher‐order cognitive mechanisms (Robertson & Baron‐Cohen, 2017).

### 4.6 Limitations

The results of the present study need to be interpreted in the context of its limitations. First, the sample size is relatively small, impacting the power of the analyses. That said, the highly significant group differences, large effect sizes, and the consistency of our main results with both the ASD phenotype and results from earlier neuroimaging studies increase the confidence in the present results. More generally, the relatively small sample size limitation is mitigated by the data‐driven, nonparametric approach to test the significance of the functional connectivity results, which provides a more rigorous and sensitive statistical test compared with parametric tests (Maris & Oostenveld, 2007) especially when the sample size is small (Warner, 2007).

## 5 CONCLUSIONS

In conclusion, our findings demonstrate significant differences in cerebro‐cerebellar functional connectivity during lexical‐semantic speech processing in ASD children. Together, the atypical pattern of both decreased and increased cerebro‐cerebellar functional connectivity is compatible with a model of atypical network organization in ASD with reduction in both network integration and network differentiation (Fishman et al., 2014; Fishman et al., 2015; Keown et al., 2017; Rudie et al., 2012; Shih et al., 2011). Finally, given the estimated directionality of the atypical cerebro‐cerebellar connectivity from the cerebellum to cortex, our results suggest that the tentative dysfunction in language processing in ASD might have a cerebellar origin, thus supporting the notion that cerebellar dysfunction could be crucial in the etiology of ASD (Becker & Stoodley, 2013; D'Mello & Stoodley, 2015; Su et al., 2021; Wang et al., 2014).

## CONFLICT OF INTEREST STATEMENT

The authors declare no conflicts of interest.

## Supporting information


**Figure S1.** Histogram of age distribution of the participants (*N* = 51).
**Figure S2.** Number of epochs per participant per condition. As epoch counts were equalized between the Speech, Jabberwocky, and Noise conditions in all analyses, only one number is shown per participant. The epoch counts did not differ significantly between the groups (Wilcoxon rank‐sum test: *z* = 0.24, *p* = .81).
**Figure S3.** ERFs in the left cerebral cortex. ROIs (left), sLORETA time courses for Speech and Jabberwocky in both groups (middle), and bar graphs of group means averaged within 100–700 ms and 1000–1500 ms (right) for (A) left temporal and (B) left parietal cortex. The ROIs are taken from Alho et al. (2021). The time courses were derived by averaging over the vertices within the ROIs. The *p*‐values are from a paired‐samples *t*‐test (two‐tailed). Error bars around the mean represent standard error of the mean.
**Figure S4.** Group difference in Speech versus Noise and Jabberwocky versus Noise coherence with right cerebellar lobule VI as seed. Bar graphs of group means with *p*‐value from two‐sample *t*‐test (two‐tailed). Coherence values were averaged within the whole spatio‐temporal cluster (see main text Figure 3a), corrected for NVIQ, and *z*‐scored. Error bars around the mean represent standard error of the mean.
**Figure S5.** Coherence between right cerebellar lobule VI and anatomical cerebral ROIs for Speech versus Jabberwocky in TD and ASD groups. (A) The anatomical ROIs (taken from FreeSurfer cortical parcellations; see main text, Section 2.8) depicted on left hemisphere inflated surface. From top to bottom: supramarginal gyrus (SMG), primary motor cortex (M1), primary auditory cortex (A1), middle frontal gyrus (MFG), inferior frontal gyrus (IFG), and middle temporal gyrus (MTG). (B) Right lobule VI seed coherence time courses from the ROIs in TD and ASD groups. Vertical dashed lines show time windows of significant group difference in the permutation test (also marked above the time windows; see main text, Section 3.2). Shaded areas around the group mean time courses indicate standard error of the mean. (C) Bar graph of group means averaged within the time windows in B with *p*‐values from two‐sample *t*‐tests (two‐tailed). Coherence values were corrected for NVIQ and the residuals were *z*‐scored. Error bars represent standard error of the mean. This figure parallels Figure 4 in the main text, that shows the same analyses, using ROIs derived from the group comparison.
**Figure S6.** Correlation of participant age with the Speech versus Jabberwocky coherence between right lobule VI and functional cerebral ROIs in both groups (TD in green, ASD in purple). (A) The functional ROIs (delineated based on group difference in the right lobule VI seed connectivity; see main text, Section 2.8) depicted on left hemisphere inflated surface. (B) Scatter plots of age against Speech versus Jabberwocky coherence using the individual peak coherence within the ROI‐specific significant group difference time windows (see main text Figure 3). (C) Scatter plots of age against Speech versus Jabberwocky coherence using the average coherence across the ROI‐specific significant group difference time windows for each individual. The Speech versus Jabberwocky coherence was corrected for NVIQ and the residuals *z*‐scored. The shaded areas around the regression line encompass the 95% confidence interval for the correlation. Correlation coefficient (*r*) and *p*‐value from Pearson correlation test (two‐tailed) for the within‐group correlations as well as Fisher *r*‐to‐*z* transformed *z*‐score and *p*‐value for the difference between the within‐group correlations are shown in each plot.

## Data Availability

All custom codes used in the processing and analyses of the data will be made available upon the acceptance of this article (https://github.com/Kenet-lab). The raw behavioral, MEG, and MRI data supporting the conclusions of this article will be made available by the authors following approval of the required Massachusetts General Hospital Data Sharing agreement.
