# Supplementary material for: Both stronger and weaker cerebro‐cerebellar functional connectivity patterns during processing of spoken sentences in autism spectrum disorder
Source: Hum Brain Mapp. 2023 Sep 9;44(17):5810–27. doi: 10.1002/hbm.26478 (PMC10619366; doi:10.1002/hbm.26478)
Supplement: Supplementary file 1 — Figure S1. Histogram of age distribution of the participants (N = 51). Figure S2. Number of epochs per participant per condition. As epoch counts were equalized between the Speech, Jabberwocky, and Noise conditions in all analyses, only one number is shown per participant. The epoch counts did not differ significantly between the groups (Wilcoxon rank‐sum test: z = 0.24, p = .81). Figure S3. ERFs in the left cerebral cortex. ROIs (left), sLORETA time courses for Speech and Jabberwocky in both groups (middle), and bar graphs of group means averaged within 100–700 ms and 1000–1500 ms (right) for (A) left temporal and (B) left parietal cortex. The ROIs are taken from Alho et al. (2021). The time courses were derived by averaging over the vertices within the ROIs. The p‐values are from a paired‐samples t‐test (two‐tailed). Error bars around the mean represent standard error of the mean. Figure S4. Group difference in Speech versus Noise and Jabberwocky versus Noise coherence with right cerebellar lobule VI as seed. Bar graphs of group means with p‐value from two‐sample t‐test (two‐tailed). Coherence values were averaged within the whole spatio‐temporal cluster (see main text Figure 3a), corrected for NVIQ, and z‐scored. Error bars around the mean represent standard error of the mean. Figure S5. Coherence between right cerebellar lobule VI and anatomical cerebral ROIs for Speech versus Jabberwocky in TD and ASD groups. (A) The anatomical ROIs (taken from FreeSurfer cortical parcellations; see main text, Section 2.8) depicted on left hemisphere inflated surface. From top to bottom: supramarginal gyrus (SMG), primary motor cortex (M1), primary auditory cortex (A1), middle frontal gyrus (MFG), inferior frontal gyrus (IFG), and middle temporal gyrus (MTG). (B) Right lobule VI seed coherence time courses from the ROIs in TD and ASD groups. Vertical dashed lines show time windows of significant group difference in the permutation test (also marked above the time windows; see m [file HBM-44-5810-s001.docx]

Supplementary Information


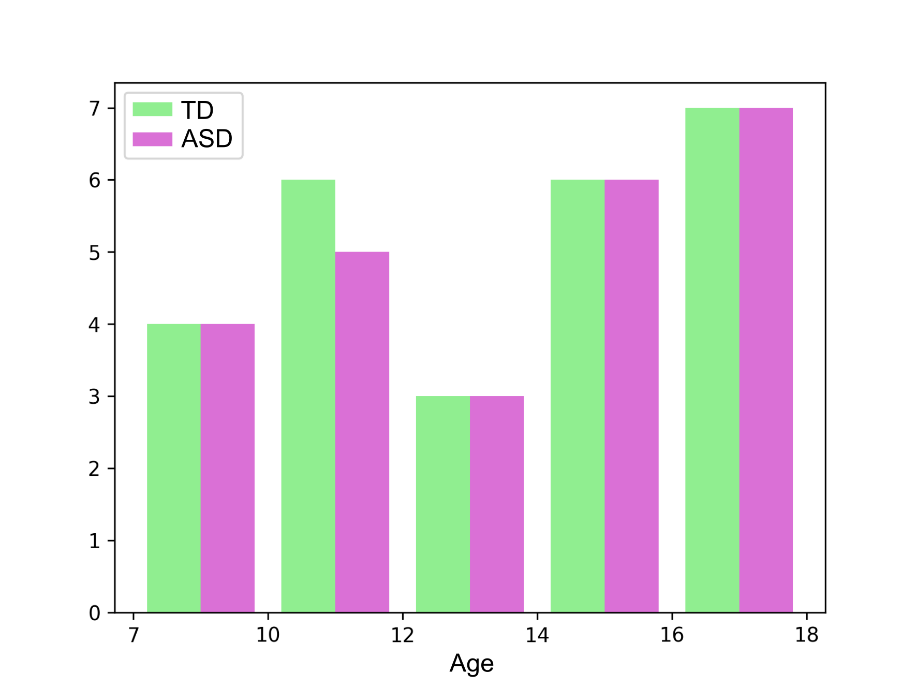


**Figure S1: Histogram of age distribution of the participants (N=51).**


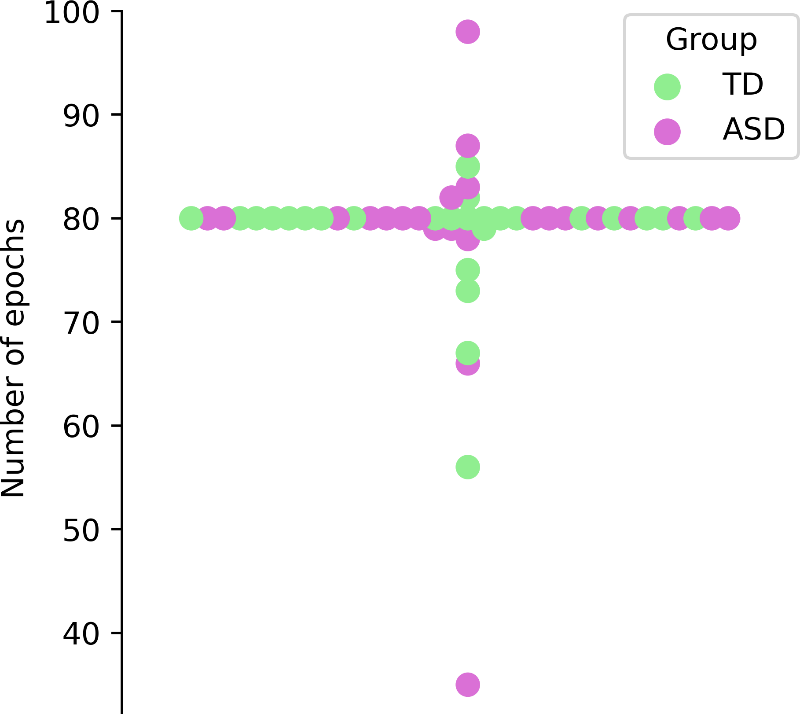


**Figure S2: Number of epochs per participant per condition**. As epoch counts were equalized between the Speech, Jabberwocky, and Noise conditions in all analyses, only one number is shown per participant. The epoch counts did not differ significantly between the groups (Wilcoxon rank-sum test: z=0.24, p=0.81).


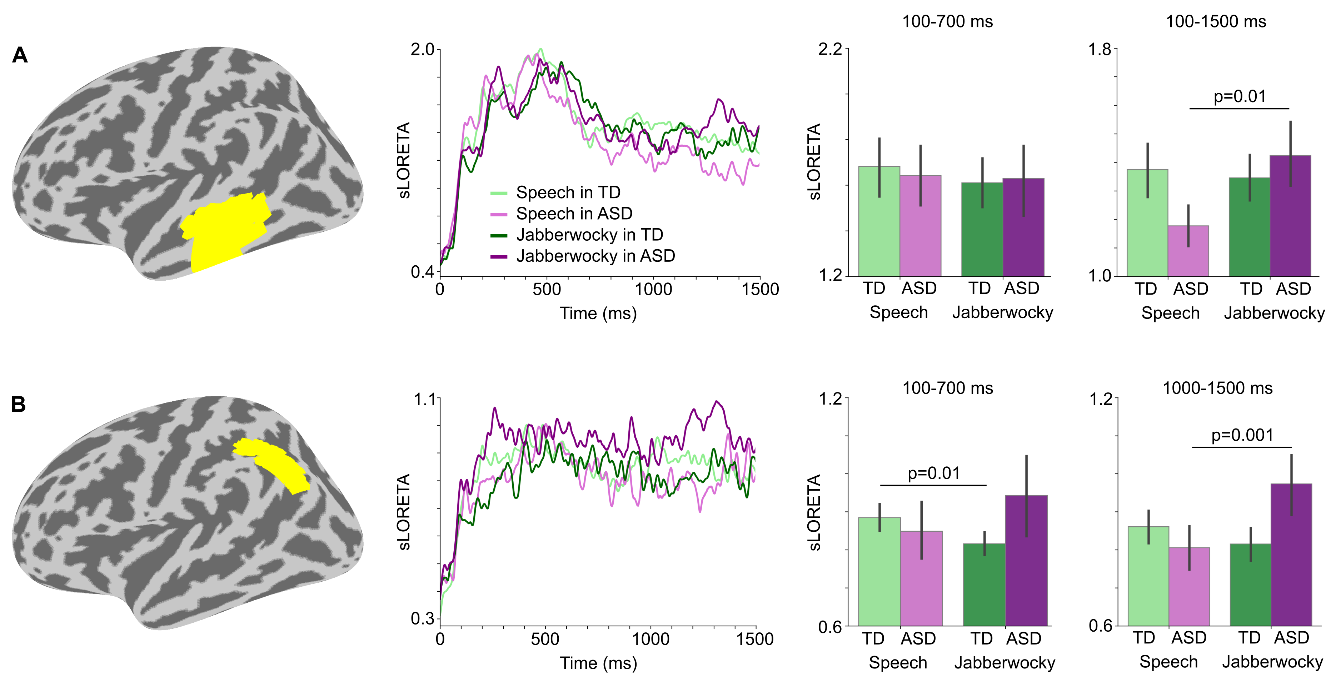


**Figure S3:** **ERFs in the left cerebral cortex**. ROIs (left), sLORETA time courses for Speech and Jabberwocky in both groups (middle), and bar graphs of group means averaged within 100-700 ms and 1000-1500 ms (right) for **A)** left temporal and **B)** left parietal cortex. The ROIs are taken from Alho et al. (2021). The time courses were derived by averaging over the vertices within the ROIs. The p-values are from a paired-samples t-test (two-tailed). Error bars around the mean represent standard error of the mean.


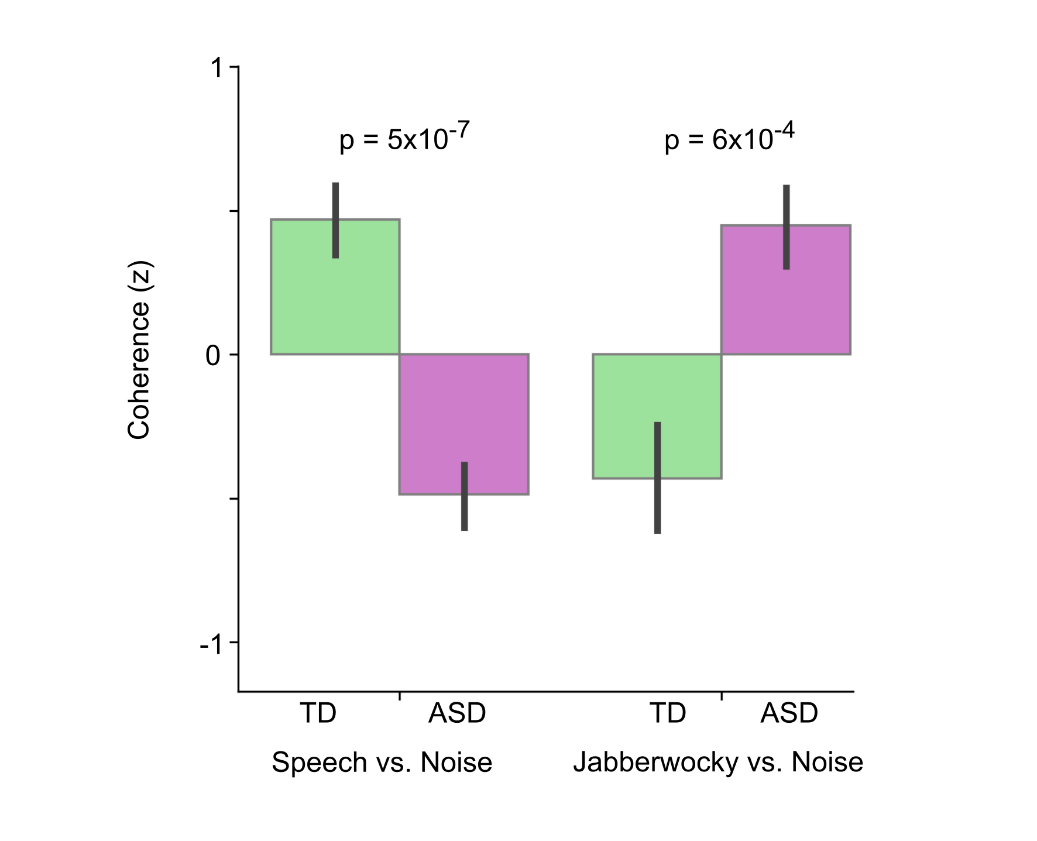


**Figure S4:** **Group difference** **in Speech vs. Noise and Jabberwocky vs. Noise coherence with right cerebellar lobule VI as seed**. Bar graphs of group means with p-value from two-sample t-test (two-tailed). Coherence values were averaged within the whole spatio-temporal cluster (see main text Fig. 3A), corrected for NVIQ, and z-scored. Error bars around the mean represent standard error of the mean.


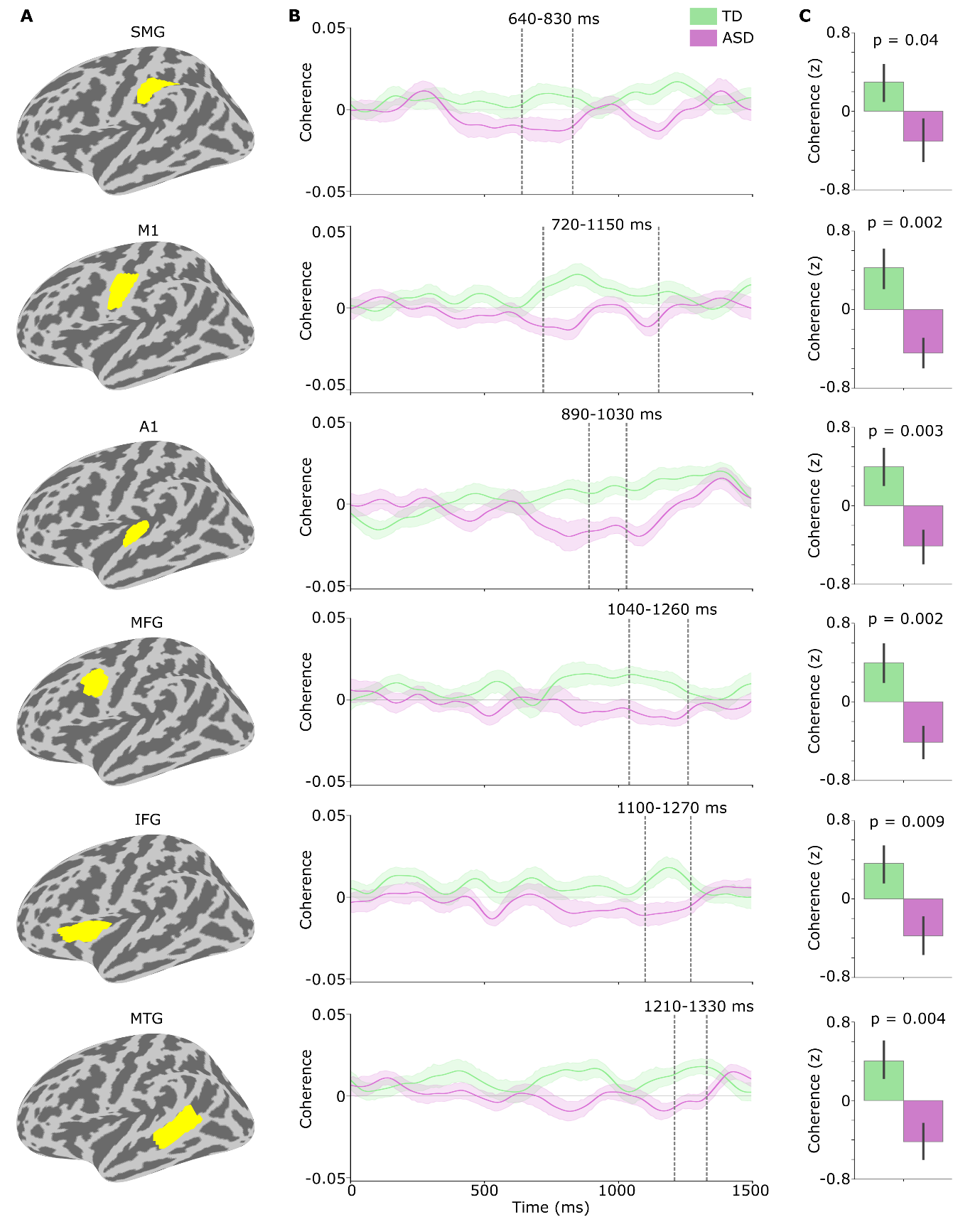


**Figure S5:** **Coherence between right cerebellar lobule VI and anatomical cerebral ROIs for Speech vs. Jabberwocky in TD and ASD groups**. **A)** The anatomical ROIs (taken from FreeSurfer cortical parcellations; see main text, section 2.8) depicted on left hemisphere inflated surface. From top to bottom: supramarginal gyrus (SMG), primary motor cortex (M1), primary auditory cortex (A1), middle frontal gyrus (MFG), inferior frontal gyrus (IFG), and middle temporal gyrus (MTG). **B)** Right lobule VI seed coherence time courses from the ROIs in TD and ASD groups. Vertical dashed lines show time windows of significant group difference in the permutation test (also marked above the time windows; see main text, section 3.2). Shaded areas around the group mean time courses indicate standard error of the mean. **C)** Bar graph of group means averaged within the time windows in B with p-values from two-sample t-tests (two-tailed). Coherence values were corrected for NVIQ and the residuals were z-scored. Error bars represent standard error of the mean. This figure parallels Figure 4 in the main text, that shows the same analyses, using ROIs derived from the group comparison.


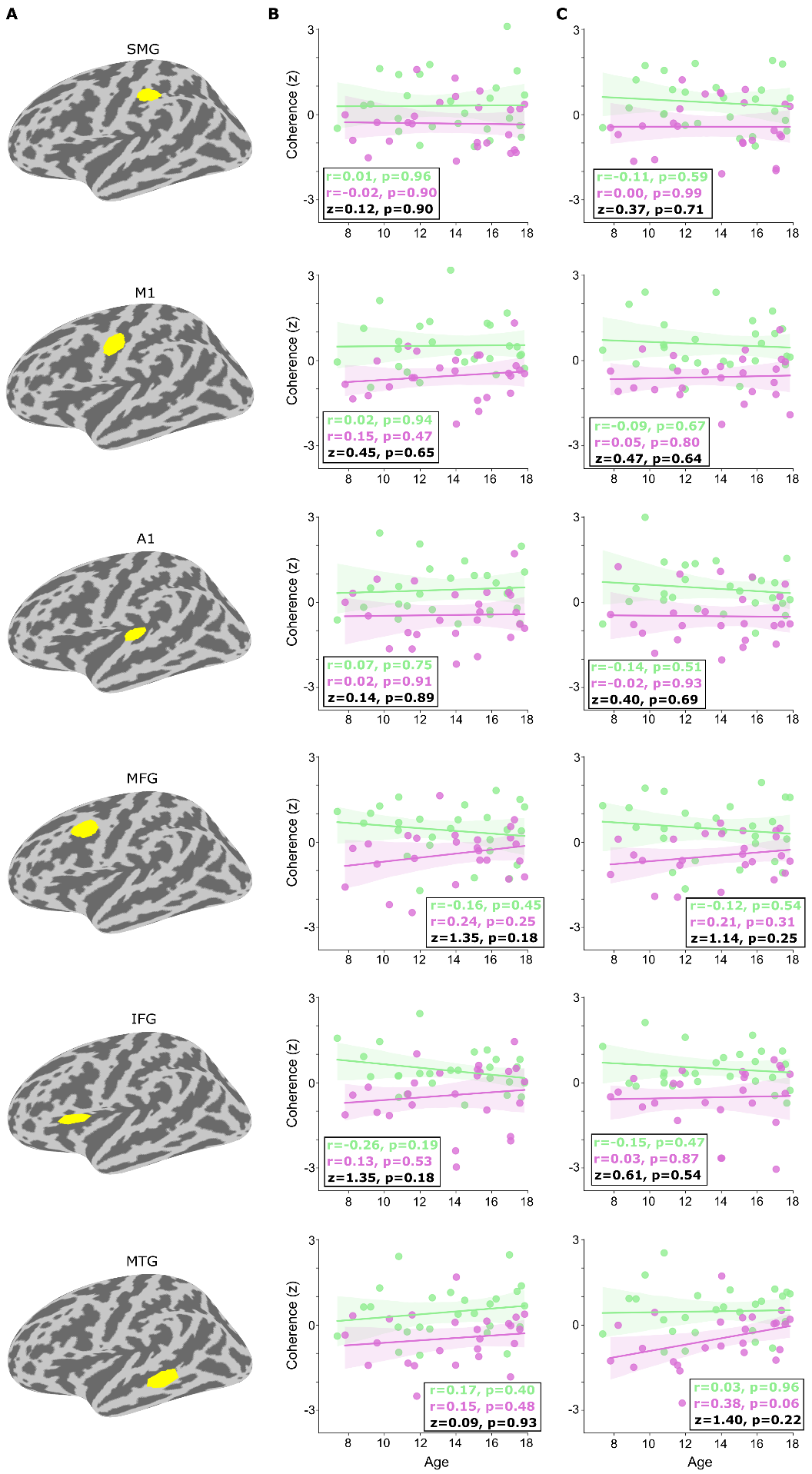


**Figure S6:** **Correlation of participant age with the Speech vs. Jabberwocky coherence between right lobule VI and functional cerebral ROIs in both groups** (TD in green, ASD in purple). **A)** The functional ROIs (delineated based on group difference in the right lobule VI seed connectivity; see main text, section 2.8) depicted on left hemisphere inflated surface. **B)** Scatter plots of age against Speech vs. Jabberwocky coherence using the individual peak coherence within the ROI-specific significant group difference time windows (see main text Fig. 3). **C)** Scatter plots of age against Speech vs. Jabberwocky coherence using the average coherence across the ROI-specific significant group difference time windows for each individual. The Speech vs. Jabberwocky coherence was corrected for NVIQ and the residuals z-scored. The shaded areas around the regression line encompass the 95% confidence interval for the correlation. Correlation coefficient (r) and p-value from Pearson correlation test (two-tailed) for the within-group correlations as well as Fisher r-to-z transformed z-score and p-value for the difference between the within-group correlations are shown in each plot.
